# Tunable, biodegradable grafting-from glycopolypeptide bottlebrush polymers

**DOI:** 10.1038/s41467-021-26808-5

**Published:** 2021-11-09

**Authors:** Zachary S. Clauss, Casia L. Wardzala, Austin E. Schlirf, Nathaniel S. Wright, Simranpreet S. Saini, Bibiana Onoa, Carlos Bustamante, Jessica R. Kramer

**Affiliations:** 1 Department of Biomedical Engineering, University of Utah, Salt Lake City, Utah 84102 USA; 2 Howard Hughes Medical Institute University of California Berkeley, Berkeley, CA 94720 USA; 3 Department of Chemistry, University of California Berkeley, Berkeley, CA 94720 USA; 4 Institute for Quantitative Biosciences, University of California, Berkeley, CA 94720 USA; 5 Molecular Biophysics and Integrated Bioimaging Division, Lawrence Berkeley National Laboratory, Berkeley, CA 94720 USA; 6 Department of Physics, University of California Berkeley, Berkeley, CA 94720 USA; 7 Department of Molecular and Cell Biology, University of California Berkeley, Berkeley, CA 94720 USA; 8 Department of Pharmaceutics and Pharmaceutical Chemistry, University of Utah, Salt Lake City, Utah 84102 USA

**Keywords:** Biopolymers, Bioinspired materials

## Abstract

The cellular glycocalyx and extracellular matrix are rich in glycoproteins and proteoglycans that play essential physical and biochemical roles in all life. Synthetic mimics of these natural bottlebrush polymers have wide applications in biomedicine, yet preparation has been challenged by their high grafting and glycosylation densities. Using one-pot dual-catalysis polymerization of glycan-bearing α-amino acid *N*-carboxyanhydrides, we report grafting-from glycopolypeptide brushes. The materials are chemically and conformationally tunable where backbone and sidechain lengths were precisely altered, grafting density modulated up to 100%, and glycan density and identity tuned by monomer feed ratios. The glycobrushes are composed entirely of sugars and amino acids, are non-toxic to cells, and are degradable by natural proteases. Inspired by native lipid-anchored proteoglycans, cholesterol-modified glycobrushes were displayed on the surface of live human cells. Our materials overcome long-standing challenges in glycobrush polymer synthesis and offer new opportunities to examine glycan presentation and multivalency from chemically defined scaffolds.

## Abstract (web summary)

Synthetic mimics of glycoproteins and proteoglycans have wide applications in biomedicine, yet preparation has been challenged by their high grafting and glycosylation densities. Here the authors show one-pot dual-catalysis polymerization of glycan-bearing α-amino acid N-carboxyanhydrides to form glycopolypeptide brushes.

## Introduction

**Fig. 1:**
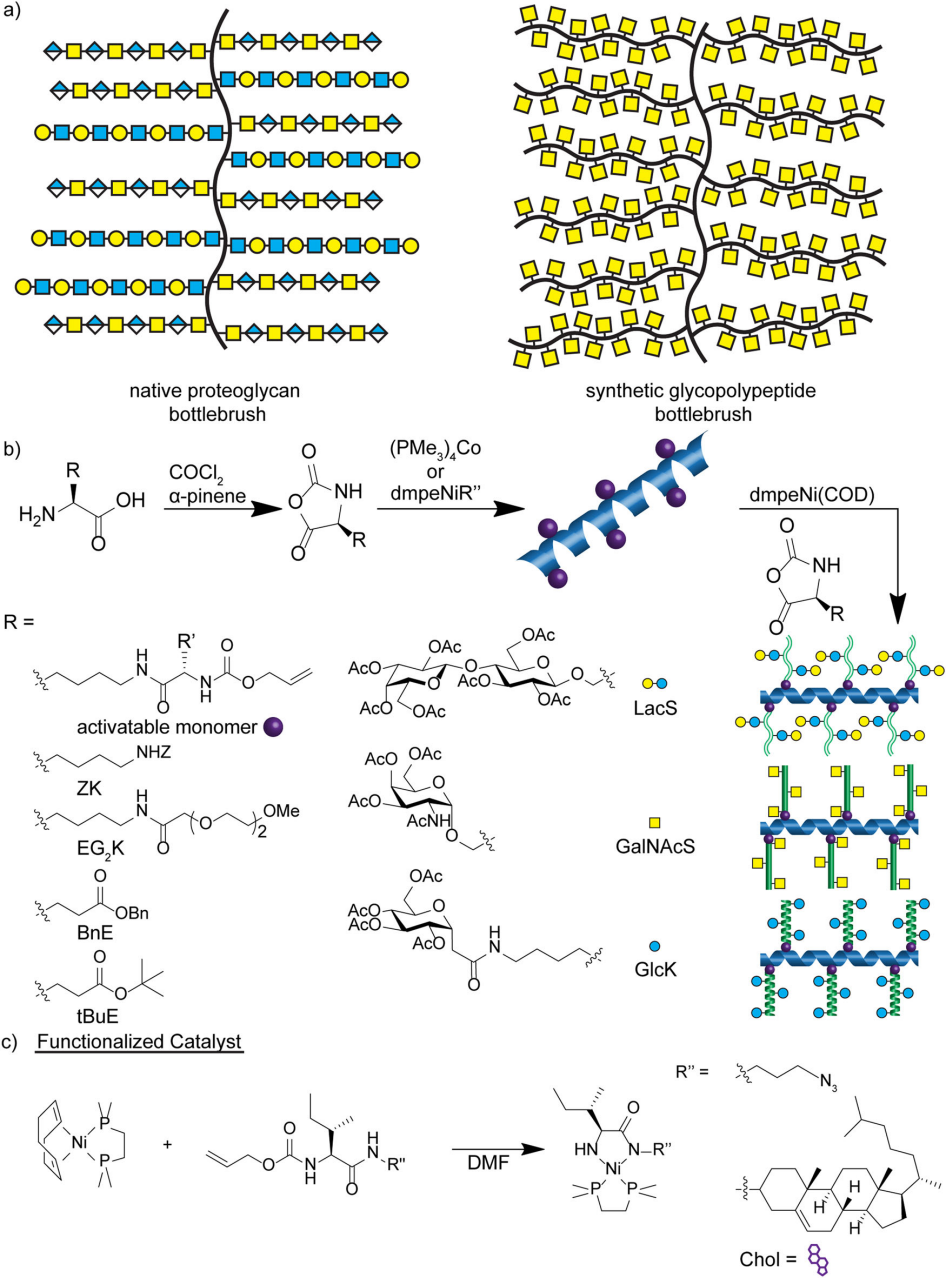
Cartoon representations of native proteoglycans vs. our synthetic glycobrushes, which are prepared via two-step, one-pot NCA polymerization. **a** Comparison of a representative native proteoglycan bottlebrush (i.e., aggrecan with polypeptide backbone and chondroitin sulfate, and keratin sulfate polysaccharide chains) with our synthetic glycobrushes. **b** Synthetic route to chemically tunable glycobrush-based dual-catalysis, one-pot NCA polymerization. Chain length, graft density, glycosylation density, and pattern are tuned via NCA monomer feed ratios and equivalents of transition metal catalysts. *R*’ = CH_2_CH_2_SCH_3_ for Met-linked AMK or CH(CH_3_)CH_2_CH_3_ for Ile-linked AIK. **c** Functional Ni^0^ catalysts used in this study to install chemical groups of interest at the chain initiation site.

The surface of all cells are densely populated with a diverse array of glycolipids and glycoproteins that collectively form the glycocalyx^1^. The extracellular matrix (ECM) is another region particularly rich in a glycoprotein subclass termed proteoglycans^2^. Both glycoproteins and proteoglycans are composed of a polypeptide backbone with enzymatically grafted saccharide chains originating predominantly at serine (Ser), threonine, and asparagine residues. The resulting structures are glycosylated molecular bottlebrushes, or glycobrushes, where saccharides are the brush bristles. Glycoproteins contain one, few, or many saccharides of diverse identity, whereas proteoglycans contain amino sugars, have regularly repeating structures, and can grow to hundreds of glycan units^3^ (Fig. 1a). Overall, these structures encompass a complex variety of molecular shapes and sizes, with variation in length and composition of the polypeptide backbone, length and graft density of the saccharide bristles, saccharide identity, and charge. These factors play essential roles in hydration, lubrication, resistance to mechanical force, and regulating diffusion of small molecules and pathogens to the cell surface^4^. Rigid, extended polypeptide backbones provide mechanical structure, whereas the saccharides bind copious water molecules and ions, as well as provide specific bioactivity^5^.

Synthetic mimics of natural glycobrushes have extensive applications in biomedicine^4^ as components of lubricants, hydrogels, and ECM mimics, and have attracted attention as bio-surrogates to probe diverse cell surface events^6,7^. Biosynthesis of proteoglycans and glycoproteins is controlled by 1000+ enzymes and yields a heterogeneous and complex mixture of structures^2^. Therefore, manipulation of individual molecular properties and study of their downstream biological effects have been hampered. Synthetic mimics offer opportunities to probe glycobiology in new ways. Glycan presentation, spacing, multivalency, and the brush architecture have all been shown to affect biological function^8,9^. For example, glycopolymer brushes have been used in arrays to detect the specificity of lectins^10,11^, have been employed in the study of viral adhesion^12^, and investigated as antimicrobial, antiviral, or antifouling agents^11^. Further, polymeric bottlebrushes are fascinating materials in their own right with properties that cannot be achieved by linear polymers^13–15^. Due their large molecular size, anisotropic conformation, and reduced chain entanglement, such materials have been investigated for application in photonic materials, films for lithographic patterning, drug delivery, tissue engineering, and tumor detection and imaging^15^. Glycans confer advantageous properties in such applications due to their non-ionic hydrophilicity and their biochemically active properties.

Here we describe a one-pot grafting-from approach to synthesize glycobrushes using α-amino acid *N*-carboxyanhydride (NCA) polymerization and dual transition metal catalysis (Fig. 1b). The method is precisely tunable within the same parameters as natural glycobrushes including polypeptide backbone composition and chain length, glyco-sidechain length and graft density, glycan identity, and chain conformation. To our knowledge, our glycobrushes are the first example of controlled polymerization of glycosylated monomers in a grafting-from approach based entirely on amino acids and saccharides.

## Results and discussion

### Design, synthesis, and analysis of tunable glycobrushes

There are three synthetic routes to bottlebrush polymers: grafting-to, where pre-existing backbone and sidechain polymers are ligated; grafting-from, where sidechain polymerization is initiated from backbone polymers; and grafting-through, where sidechain polymers act as macromonomers to yield the backbone chain^13,16^. A general challenge facing all syntheses of bottlebrush polymers is steric hindrance at the backbone due to dense grafting, resulting in low molecular weight (MW) chains and low grafting efficiencies. Compared to grafting-through and grafting-to strategies, the grafting-from method offers improved alleviation of steric hindrance due to the gradual growth of the sidechains. Synthesis of glycobrushes has focused mainly on the grafting-to approach^8,9,17–19^, which is convenient, as both backbone and sidechains can be individually synthesized and characterized. However, this method typically suffers from low graft density and incomplete functionalization. Although much beautiful work has been done to optimize grafting-from systems utilizing controlled radical, ring-opening metathesis, and NCA polymerizations^13,16,20–22^, there are few reports on the use of glyco-monomers and prior work has focused exclusively on reversible deactivation radical polymerization^23–28^. This method yields hydrocarbon backbone polymers incapable of the hydrogen bonds crucial to protein conformation and function, requires non-native glycan structures, and, to date, initiation inefficiency has limited the grafting density, as only low MW sidechains were produced. In addition, such materials would not be substrates for natural proteases and their degradation products would yield biologically foreign materials.

Inspired by the work of Rhodes and Deming^29^ who synthesized bottlebrushes based entirely on polypeptides, we sought to develop tunable glycobrushes based entirely on amino acid and saccharide building blocks, which we expect to be biodegradable^30^. Toward this end, we employed two-step NCA polymerization in a one-pot dual-catalysis system to form glycobrushes with graft density up to 100% if desired, high MW chains, and tunable sidechain morphology and composition. For the backbone polypeptide, an allyloxycarbonyl (Alloc) functionalized lysine (Lys) NCA was utilized, as the Alloc group is inert to established Co^0^ polymerization initiators but reacts with electron-rich Ni^0^ species to form amido-amidate nickelacycles^29,31,32^ (Fig. 1b). These nickelacycle species are known initiators of NCA polymerization and served as sites for growth of glycopolypeptide branches. Isoleucine (Ile) or methionine (Met) were used as linkers between the Alloc and Lys, to generate two activatable monomers Alloc-Met-Lys (AMK) NCA and Alloc-Ile-Lys (AIK) NCA. We chose Ile, as it confers good organic solubility, and Met, as peptide bonds can be selectively cleaved at Met sites using cyanogen bromide (CNBr). This feature liberates the sidechains from the backbone, enabling separate analyses of their properties. Therefore, most initial polymerization studies were conducted with AMK NCA. In addition, Met residues could later serve as substrates for selective bioconjugation of desired functional molecules^33,34^ or as a site of oxidation to manipulate backbone hydration^35,36^.

Graft density was readily modulated by stoichiometric copolymerization of activatable AMK or AIK NCAs and non-activatable NCAs (Table 1 and Fig. 1b). For non-activatable NCAs, we chose *N*-ε-carbobenzyloxy-l-Lys (ZK), γ-benzyl-l-glutamate (BnE), γ-*tert*-butyl-l-glutamate (tBuE), and diethylene glycol functionalized Lys (EG_2_K) NCAs. We chose these, as ZK, BnE, and tBuE are well-established monomers for NCA polymerization and as non-ionic EG_2_K requires no deprotection chemistry and offers both aqueous and organic solubility. In addition, polymers of these structures are all known to form α-helices^37,38^, resulting in ordered display of the Alloc groups. NCAs were polymerized in various relative ratios from 25% to 100% AMK, and at varied monomer to initiator (M : I) ratios with (PMe_3_)_4_Co initiator at room temperature in tetrahydrofuran (THF). As expected, polymer MWs were higher than predicted from monomer to initiator stoichiometry, which is due to the known incomplete efficiency of (PMe_3_)_4_Co initiation in THF^39^. High MW homo- or co-polypeptide AMK or AIK backbones up to degrees of polymerization (DPs) ca. 300, could typically be formed within 1–2 h.

**Table 1:** Representative polymerization data for tunable glycobrushes.

		Backbone		Brush			Cleaved branches	
Entry	Name	[M] : [I]^a^	*M*_*n*_^b^	*M*_*n*_^b^	*Ð*^b^	[M] : [I]^c^	*M*_*n*_^b^	*Ð*^b^
1	PAMK_63_-*g*-PGalNAcS_33_	20	21.6k	434k	1.43	20	13.7k	1.28
2	PAMK_63_-*g*-PLacS_43_	20	21.6k	565k	1.40	20	30.0k	1.02
3	PAMK_63_-*g*-PGlcK_17_	20	21.6k	557k^d^	ND	10	8.5k	1.08
4	PAMK_63_-*g*-PGlcK_23_	20	21.6k	750k^d^	ND	20	11.4k	1.18
5	PAMK_63_-*g*-PGlcK_117_	20	21.6k	3686k^d^	ND	100	58.5k	1.03
6	PAMK_63_-*g*-(PGlcK_0.5_-*s*-PBnE_0.5_)_24_	20	21.6k	566k^d^	ND	10	8.6k	1.05
7	PAMK_63_-*g*-(PGlcK_0.5_-*s*-PBnE_0.5_)_38_	20	21.6k	884k^d^	ND	20	13.7k	1.15
8	PAMK_63_-*g*-(PGlcK_0.5_-*s*-PBnE_0.5_)_54_	20	21.6k	1246k^d^	ND	50	19.4k	1.28
9	PAMK_63_-*g*-(PGlcK_0.5_-*s*-BnE_0.5_)_82_	20	21.6k	1881k^d^	ND	100	29.5k	1.22
10	(PAMK_0.25_-*s*-PZK_0.75_)_75_-g-PGlcK_68_	20	21.2k	661k^d^	ND	100	34.2k	1.19
11	(PAMK_0.5_-*s*-PEG_2_K_0.5_)_150_-g-PGlcK_35_	50	47.3k^e^	1729k^d,e^	ND	100	22.5k	1.04
12	(PAMK_0.5_-*s*-PEG_2_K_0.5_)_300_-g-PGlcK_45_	100	94.6k^e^	3007k^d,e^	ND	100	19.5k	1.09

^a^Molar ratio of backbone NCA monomer to (PMe_3_)_4_Co initiator.

^b^Observed *M*_*n*_ and *Ð* after polymerization as determined by GPC/LS and ^1^H NMR.

^c^Molar ratio of branch NCA monomers to macroinitiator active sites.

^d^Calculation of *M*_*n*_ from separate GPC/LS analysis of branch and backbone polymers.

^e^Theoretical *M*_*n*_.

The Alloc-containing polypeptides were then directly treated with 1,2-bis(dimethylphosphino)ethane nickel cyclooctadiene (dmpeNi(COD)) at 80 °C in dimethylformamide (DMF), to generate amido-amidate nickelacycle-initiating groups along the backbones. These macroinitiators were combined with various glycan-bearing NCAs to form tunable glycobrushes. We chose Ser (S) NCAs bearing α-*N*-acetylgalactosamine (GalNAcS) or disaccharide β-lactose (LacS), as these native structures play important roles in cell surface biology^1^. Polymers of these two structures differ in that poly(GalNAcS) forms highly rigid rods^40^, whereas poly(LacS) forms disordered structures^41^. To expand the scope of glycans presented and to investigate the role of conformation in sidechain growth, we included α-glucose-modified Lys (GlcK) NCAs, which are known to form α-helical structures when polymerized^42^. GlcK is also attractive, as it features an anomeric C-linkage that has been shown to bind biological targets with affinities equal to O-linkages, but that is resistant to acidic hydrolysis and enzymatic deglycosylation^43–45^. Similar to modulation of backbone composition, sidechain glycosylation density was readily modulated by stoichiometric incorporation of BnE NCAs (Table 1, entries 7–9).

**Fig. 2:**
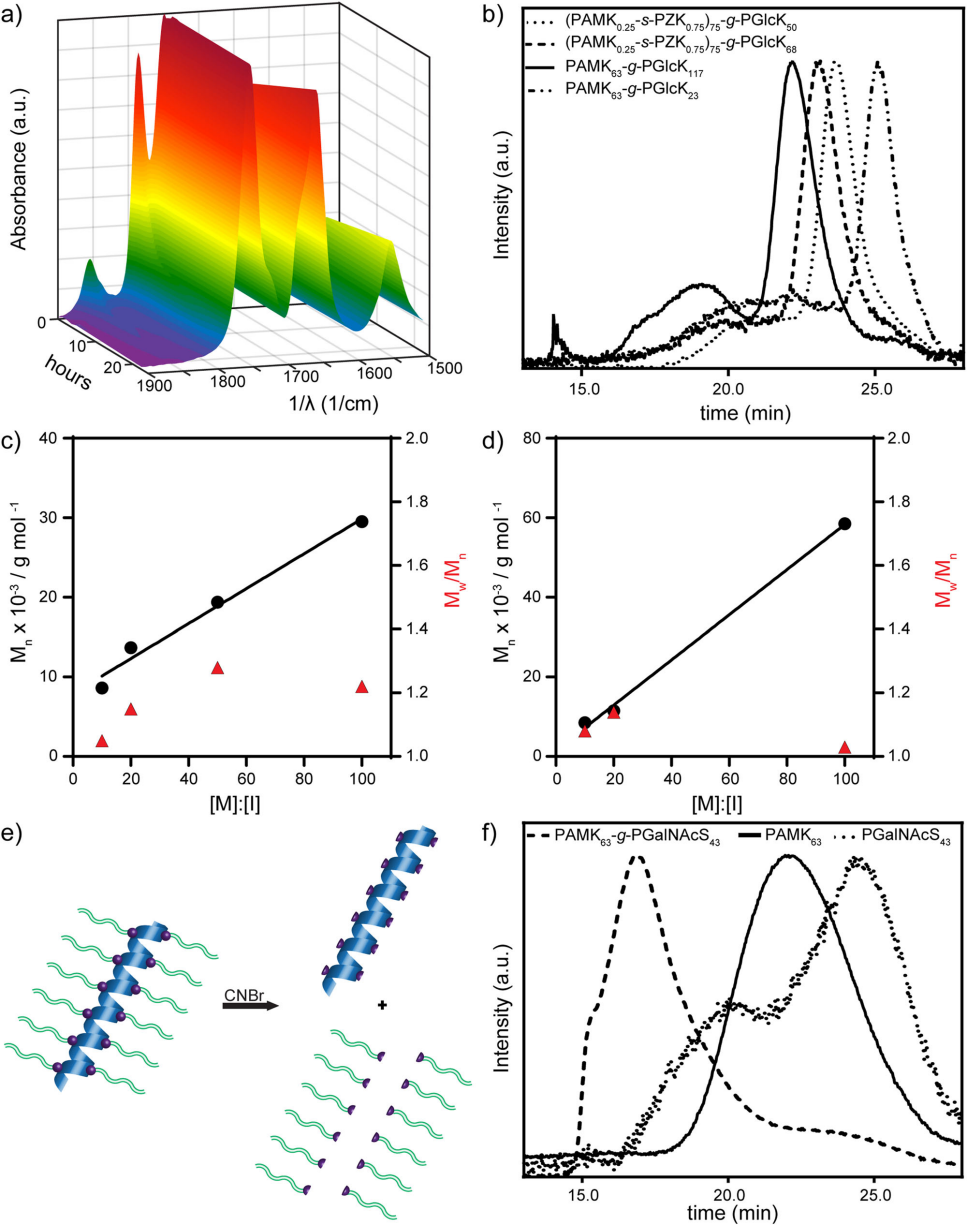
Glyco-NCAs are quantitatively converted to glycobrush polypeptides via controlled and living polymerization. **a** ATR-FTIR traces indicating polymerization of GlcK NCA from PAMK_63_ macroinitiator to form PAMK_63_-*g*-PGlcK_117_ glycobrushes. **b** Representative GPC/MALS/RI analysis of glycobrushes with varied graft density and chain lengths. **c**, **d** Data from GPC/MALS/RI analysis of sidechains cleaved from 100% graft density glycobrushes, *M*_*n*_ (number average molecular weight, black circles) and ***Ð*** (weight average molecular weight × number average molecular weight^−1^, *M*_*w*_/*M*_*n*_, red triangles), **c** is 50% glycosylated sidechains 1 : 1 GlcK:BnE, and **d** is 100% glycosylated GlcK sidechains. **e** Cleavage of glycopolypeptide branches at Met residues enables separate analysis of glycobrushes and branches. **f** Representative example GPC/MALS/RI analysis of a PAMK_63_ backbone, 100% grafting density, and 100% glycan density glycobrush, PAMK_63_-*g*-PGalNAcS_43_ glycobrush, and cleaved PGalNAcS_43_ branches.

Polymerization reactions were monitored by attenuated total reflectance Fourier transform infrared spectroscopy (ATR-FTIR) via observation of NCA absorbances at ca. 1850 and 1790 cm^−1^ vs. polypeptide absorbances at ca. 1650 and 1540 cm^−1^ (Fig. 2a). After complete conversion of NCA to glycobrush, polymers were analyzed by a combination of ^1^H nuclear magnetic resonance (NMR) and gel permeation chromatography coupled with tandem 18-angle light scattering and refractive index analysis (GPC/MALS/RI). For ^1^H NMR, polypeptide N termini were reacted with highly monodisperse poly(ethylene glycol) (PEG)-isocyanates and the PEG was used as an internal standard for MW calculations^46^. See the Supporting Information (SI). For GPC/MALS/RI in 0.1 M LiBr in DMF, aliquots of polymerization reactions were directly injected. Activation of Alloc groups by dmpeNi(COD) was previously shown to be essentially quantitative^29^ and, indeed, we observed disappearance of Alloc peaks by ^1^H NMR. However, to ensure that glyco-NCA polymerization and brush growth was due to activated AMK or AIK rather than any excess free Ni^0^, we activated only 95% of the available Alloc groups and no free chains were observed by GPC/LS.

**Fig. 3:**
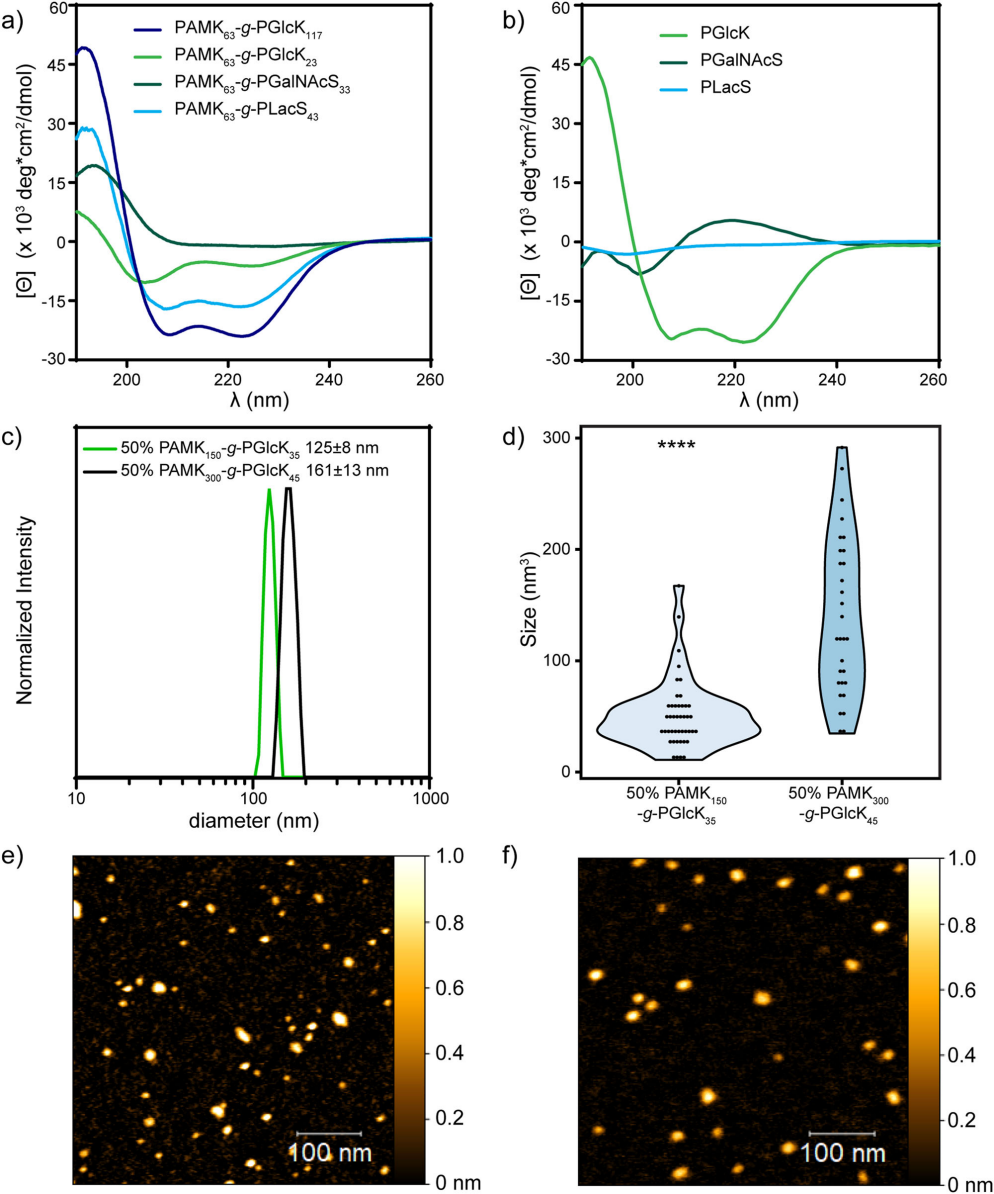
Analyses of glycobrush conformations and morphologies. **a** CD spectra of various glycobrushes. **b** CD spectra of linear glycopolypeptide branches. **c** Hydrodynamic size distribution determined via DLS. **d** Glycobrush particle volume analysis from AFM images. ****P-value < 0.0001 from a two-sided Mann–Whitney test. *n* = 30 for (PAMK_0.5_-*s*-PEG_2_K_0.5_)_300_-g-PGlcK45 and *n* = 44 for (PAMK_0.5_-*s*-PEG_2_K_0.5_)_150_-g-PGlcK_45_. **e** AFM image of (PAMK_0.5_-*s*-PEG_2_K_0.5_)_150_-g-PGlcK_35_. **f** AFM image of (PAMK_0.5_-*s*-PEG_2_K_0.5_)_300_-g-PGlcK_45_.

We were delighted to observe quantitative conversion of monomer to polymer with linear and predicable glycopolypeptide sidechain growth even at 100% graft density (Table 1, Entries 1–9). Remarkably, sidechains of 100% glycosylation density could be grown up to ca. 117 residues. Clear shifts in MW were observed by GPC/MALS/RI analysis for glycobrushes prepared with varied M : I ratios and at varied graft densities (Fig. 2b and SI Fig. 10). For some high MW samples with 100% branch density, we observed slightly viscous DMF solutions. GPC/MALS/RI analysis revealed a MALS shoulder on the elution peak, indicating the presence of some aggregates in organic solvent. As even very low-concentration aggregates can strongly scatter light^47–49^, we examined the RI trace of these samples. RI analysis indicates very little mass in the region we attribute to aggregation, which rationalizes the low calculated dispersities (see SI for representative RI trace). We did not observe such aggregation for lower graft density glycobrushes, nor the backbones themselves (Fig. 2f and SI Fig. 10). Further, no aggregation was observed for 50% grafting density brushes after deprotection and dissolution in aqueous solution as evidenced by dynamic light scattering (DLS) (Fig. 3c).

However, we still wondered whether minor aggregation or other factors could result in inconsistent growth of sidechains for 100% graft density brushes. Therefore, we desired analysis of the sidechains separated from the backbone. To achieve this, AMK-based glycobrushes were treated with CNBr to cleave the branches from the backbone at the Met sites (Fig. 2e) and the cleavage reaction was directly analyzed by GPC/MALS/RI. Figure 2f is a representative GPC/MALS/RI trace of the backbone, a glycobrush, and cleaved branches. We again observed very low levels of aggregates, which had negligible mass by RI (see SI for representative RI traces). This we attribute to backbone structures, as we directly analyzed aliquots of the branch cleavage reaction. Figure 2c indicates linear sidechain growth for 50% glycosylated sidechains and Fig. 2d indicates linear sidechain growth of 100% glycosylated sidechains, and remarkably low dispersities ranging from 1.03 to 1.28. Both samples are 100% grafting density where every backbone monomer unit has a sidechain. Table 1, Entries 1–9, contains the polymer data for these samples (see the SI for additional GPC data, SI Fig. S10). Previously reported atom transfer radical polymerization grafting-from glycobrushes suffered from inefficient initiation ranging from 23% to 38%, necessitated monomer conversions of <11%, and used non-native glycans and polymer backbones^23–26^. Our system overcomes these challenges offering quantitative initiation and conversion, up to 100% graft density, complete tunability in chain length, glycan identity and density, native components, and MWs on par with native proteoglycan and glycoprotein structures.

### Kinetics of glycobrush growth

During the course of this work, we observed a striking rate acceleration for growth of GlcK brushes as compared to GalNAcS and LacS brushes. Conversion from monomers to glycobrushes were monitored by ATR-FTIR as previously described and data were normalized to the absorbance intensities of acetate functional groups that remain constant throughout the course of the reactions (see SI Figs. S1–9). Remarkable 20- and 59-fold rate enhancements were observed for polymerization of GlcK brushes as compared to GalNAcS and LacS, respectively (Table 2). We believe this phenomenon is due to the resulting secondary structure of the branches where polyGlcK forms α-helices, whereas polyGalNAcS is extended and rod-like and polyLacS forms disordered structures. Prior work from Cheng and colleagues^50^ indicated rate acceleration for growth of neighboring α-helical polymers due to cooperative interactions of macrodipoles (vide infra). To confirm these secondary structures, we performed conformational analyses of glycobrushes and linear glycopolypeptides by circular dichroism (CD) spectroscopy. As expected from previously reported data on GlcK homopolymers^42^, strong negative absorbances at 208 and 222 nm were observed for both linear chains and GlcK brushes, indicating classical α-helices (Fig. 3a, b). GalNAcS linear polymers and brushes display a positive maxima at 218 nm, negative minima at 202 nm, and positive maxima at 194 nm, which indicate a uniquely rigid, extended structure similar to a polyproline helix where the glycosyl amide hydrogen bonds to the peptide backbone^40^. For the LacS linear polymers, we observed a fairly weak absorbance and minima at 198. This CD pattern likely indicates a disordered structure. The CD of LacS glycobrushes is dominated by the strong absorbance of the helical polypeptide backbone.

**Table 2:** Second-order kinetic parameters for NCA polymerization and growth of glycopolypeptide branches with various secondary structures and at varied graft densities.

Polymer	*k*_2_ (M^−1^ h^−1^)	Branch conformation
PAMK_63_-*g*-PGlcK_117_	823	
(PAMK_0.25_-*s*-PZK_0.75_)_75_-*g*-PGlcK_50_	1120	
(PAMK_0.25_-*s*-PZK_0.75_)_75_-*g*-PGlcK_68_	1920	
PGlcK_99_	3939	
PAMK_63_-*g*-PGalNAcS_33_	14	
PAMK_63_-*g*-PLacS_43_	41	
(PAMK_0.25_-stat-PZK_0.75_)_75_-*g*-PLacS_25_^*^	25	

*DP is the M:I used to calculate k_2_.

In the work by Cheng and colleagues^50^, polynorbornene macroinitiators bearing varied densities of trimethylsilylamine groups were used to initiate polymerization of BnE NCA into helical polyBnE bottlebrushes. They reported a dramatic rate enhancement for growth of the neighboring α-helical polymers. Linear polymer chains will likely grow as individual units in solution, but by comparison, brush sidechains will grow in a sterically crowded environment where they can interact. Cheng and colleagues^50^ rationalized the growth rate acceleration of helix-forming brush sidechains as compared to disordered chains, due to the cooperative interactions of helix macrodipoles. Although the polymerization chemistry and backbone/branch polymer structures are very different than those we describe here, we observed the same phenomena. Steric effects are also a probable factor in the kinetics we observed, as reducing graft density to 25% increased the speed of polyGlcK brush growth 2.3-fold. However, considering that GalNAcS and GlcK are both monosaccharide-bearing structures, the 59-fold rate enhancement for 100% density GlcK vs. GalNAcS brushes is most likely a conformational effect rather than a steric effect. Polymerization kinetics of GalNAcS and several other glycoS NCAs were reported previously, and was determined to be essentially the same as a modified Lys NCA^40,41^, so kinetics of individual NCAs are not a likely source of such dramatic rate enhancements. We believe we have now independently confirmed the effect of cooperative interactions of α-helical macrodipoles in neighboring polymers in our transition metal-catalyzed system with glycobrushes.

### Aqueous morphology

Our glycobrushes were readily deacetylated by treatment with K_2_CO_3_ in methanol/water to yield water-soluble glycobrushes with native glycans. To characterize the conformation and morphology of our glycobrushes, we analyzed 50% graft density GlcK glycobrushes with varied sidechains and backbone DPs by DLS and atomic force microscopy (AFM). DLS of (PEG_2_K_0.5_-*s*-PAMK_0.5_)_150_-*g*-PGlcK_35_ and (PEG_2_K_0.5_-*s*-PAMK_0.5_)_300_-*g*-PGlcK_45_ revealed uniform hydrated particle sizes of 125 ± 8 nm and 161 ± 16 nm, respectively (Fig. 3c). AFM imaging of the glycobrushes was performed on freshly cleaved mica in tapping mode. Similar to native proteoglycans, our structures were prone to intermolecular association, so low-concentration 5 nM solutions were utilized to enhance observation of single molecules. Representative images of (PEG_2_K_0.5_-*s*-PAMK _0.5_)_150_-*g*-PGlcK_35_ and (PEG_2_K_0.5_-*s*-PAMK_0.5_)_300_-*g*-PGlcK_45_ are shown in Fig. 3e, f. As expected from the DLS data, we observed spherical and ellipsoid particles. Based on peptide bond lengths, we had estimated the ideal dimensions of (PEG_2_K_0.5_-*s*-PAMK _0.5_)_150_-*g*-PGlcK_35_ as 23 × 15 nm and (PEG_2_K_0.5_-*s*-PAMK_0.5_)_300_-*g*-PGlcK_45_ as 46 × 15 nm. Resolution of less than ca. 15 nm is challenging due to the convolution of the AFM tip; however, we did observe a clear statistical difference in particle sizes from AFM images of 150mer vs. 300mer backbone glycobrushes with similar sidechain lengths as indicated in the violin plots shown in Fig. 3d. All particles deemed aggregates were excluded and only volumes of single particles were included in the analysis. Particle volumes expected by conversion of DLS-obtained diameters (*V* = 4*πr*^3^) are larger than those observed by AFM, but this is most likely due to the hydration of the glycobrushes in aqueous solution vs. dehydration and kinetic trapping on the mica surface.

### Biodegradation and toxicity assays

**Fig. 4:**
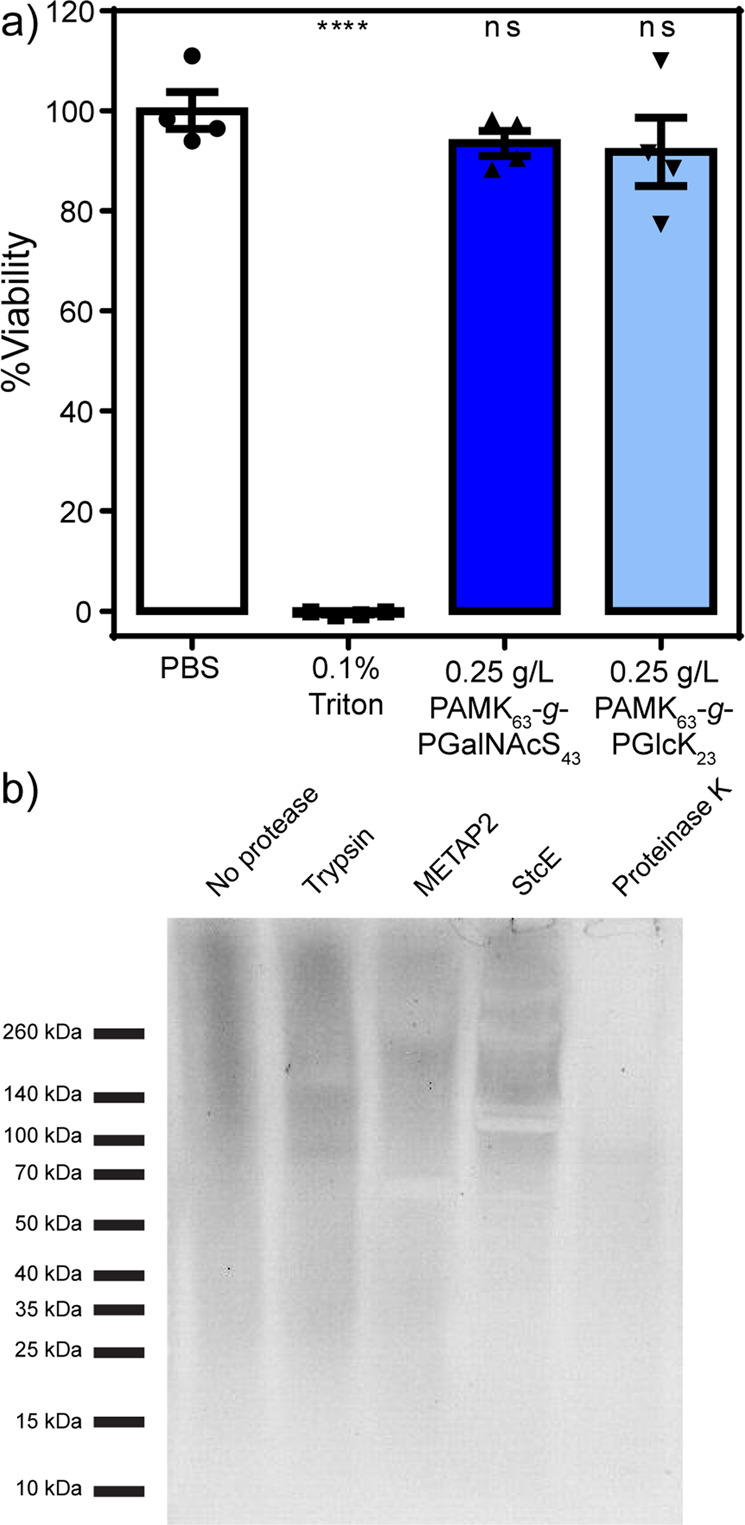
Glycobrushes are non-toxic to live human cells and can be slowly degraded by natural proteases. **a** Cytotoxicity in HEK293T cells after 24 h incubation with PAMK_63_-*g*-PGlcK_23_ or PAMK_63_-*g*-PGalNAcS_33_ (CCK-8 assay). Data are presented as mean ± SEM. One-way ANOVA adjusted for multiple comparisons was used to compare each treatment to the PBS control. “ns” indicates not significant with α-level of 0.05 and ****P-value < 0.0001 when compared to the PBS control. *P*-value = 0.5665 and 0.3909 for PAMK_63_-*g*-PGalNAcS_33_ and PAMK_63_-*g*-PGlcK_23_, respectively. **b** Polyacrylamide gel of glycobrush PAMK_63_-*g*-PGalNAcS_33_ or glycobrush after treatment with various proteases. Digestions were performed at 37 °C for 48 h. Gels were imaged using a periodate-based stain. Studies were performed in four replicates.

To investigate the utility of our glycobrushes as surrogates for native proteoglycans, we examined their effect on cell viability, their biodegradability, and their ability to be displayed on the surface of live cells. A commercial Cell Counting Kit-8 (CCK-8) colorimetric assay for the determination of cell proliferation and cytotoxicity was conducted in human embryonic kidney cells (HEK293T) after 24 h treatment with glycobrushes bearing either GalNAcS or GlcK. The phosphate-buffered saline (PBS) control was normalized to 100% and other samples are reported relative to that value. Triton was used as a positive control to kill cells and ensure functional assay conditions. A one-way analysis of variance test was conducted and compared to controls, revealing our glycobrushes had no statistically significant effect on viability (Fig. 4a).

As our materials are composed entirely of sugars and amino acids, we explored their susceptibility to natural proteases. We selected a sample with 100% graft density and 100% glycosylation, PAMK_63_-*g*-PGalNAcS_33_, as this structure is quite sterically hindered. If this sample can be digested by proteases, then we can safely extrapolate that our lower graft and glycosylation density samples will as well. Previous research on related linear GalNAcS glycopolypeptides indicated that this material is a substrate for both a general protease (trypsin) and a glycoprotein-specific protease (secreted protease of C1 esterase inhibitor)^30^, so we included these alongside general protease Proteinase K and Met-specific protease Met aminopeptidase 2 (MetAP2). Over a 48 h time period, we observed minor degradation by trypsin, MetAP2, and StcE. Proteinase K treatment, however, resulted in nearly complete degradation of the glycobrush (Fig. 4b). Although a full study of the degradation properties of various brush compositions is outside the scope of this work, we have demonstrated that these materials are protease resistant but are substrates for natural proteases and will eventually degrade.

### Glycocalyx engineering

**Fig. 5:**
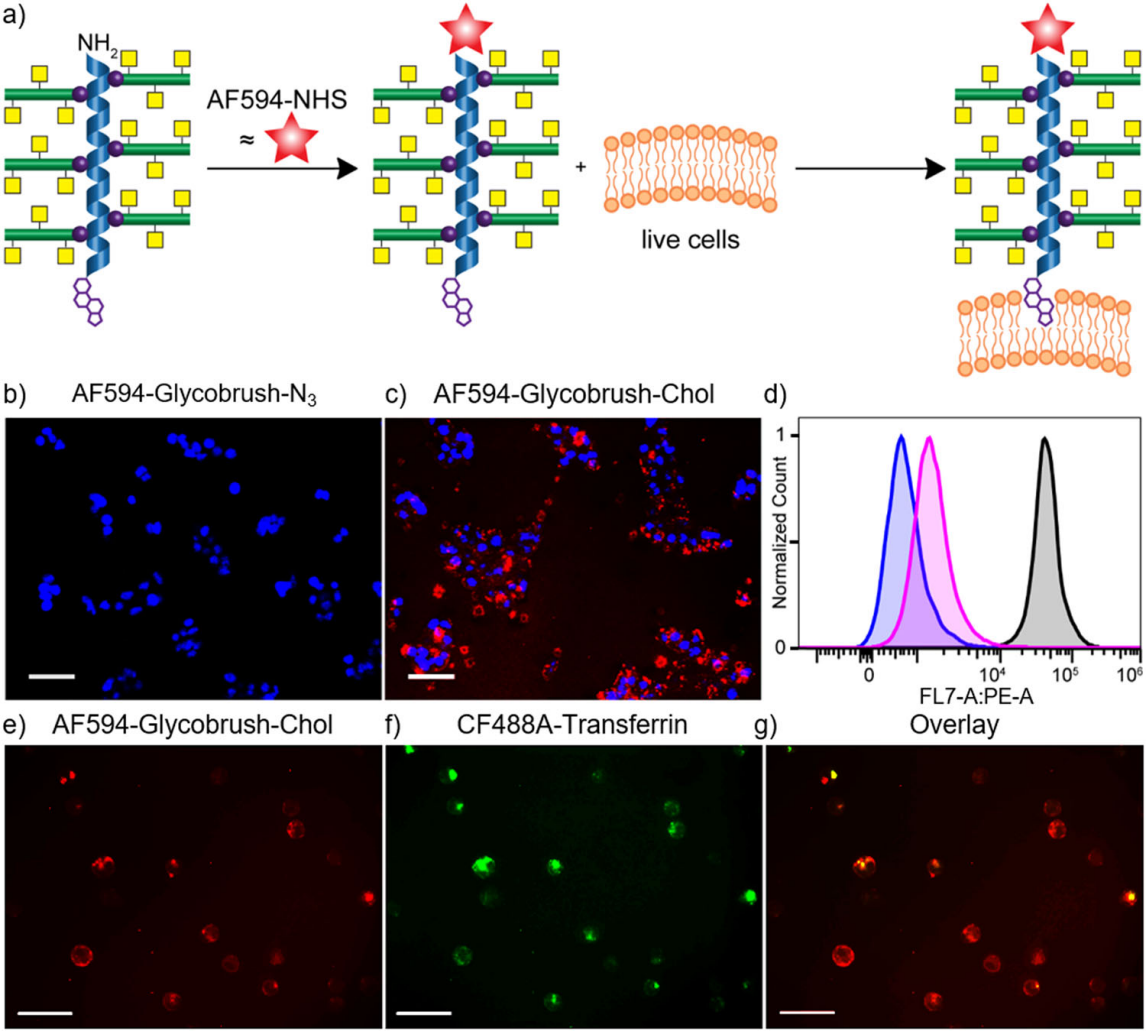
Robust and prolonged glycocalyx engineering using Chol-terminal GalNAcS- glycobrushes. **a** Reaction scheme for conjugation of AF594-NHS to glycobrush (PLG_0.8_-*s*-PAIK_0.2_)_50_-*g*-PGalNAcS_13_-Chol amino termini, followed by engineering of the glycocalyx of live epithelial cells. Cell images in **b**, **c** are 24 h after incubation **b** with 15 µM AF594-glycobrush-N_3_ or **c** with 15 µM AF594-glycobrush-Chol. **d** Flow cytometry data of HEK293T cells untreated (blue) or treated with AF594-glycobush-N_3_ (magenta) or AF594-glycobrush-Chol (black). Cell images in **e**–**g** are paraformaldehyde-fixed HEK293T cells that were incubated with 15 µM AF594-glycobrush-Chol and 30 μg/mL CF488A-transferrin, where **e** is imaging for AF594-glycobrush-Chol, **f** is imaging for CF488A-transferrin, and **g** is the overlay of the images in **e** and **f**. Scale bars are 50 µm. Images in **b**, **c**, **e**–**g** are representative of four separate experiments.

A subset of native proteoglycans are directly tethered to the cell surface through lipid anchors^51^. In a biomimetic strategy, we utilized terminal cholesterol groups to tether our glycobrushes to the surface of live human epithelial cells (Fig. 5a). This group was installed on every chain end via our functional Ni initiators. The cholesterolamide structure (Chol) was chosen based on a report indicating prolonged cell surface residence time of attached cargo as compared to linear lipids due to pooling inside of endosomes and recycling back to the surface^52^. We conjugated AF594-NHS to the brush amino termini for visualization. Live human epithelial cells (HEK293T) were incubated with 15 µM solutions of glycobrushes containing either the terminal Chol residue, AF594-(PLG_0.8_-*s*-PAIK_0.2_)_50_-*g*-PGalNAcS_13_-Chol, or a control glycobrush of the same composition but containing a terminal azide residue, AF594-(PLG_0.8_-*s*-PAIK_0.2_)_50_-*g*-PGalNAcS_13_-N_3_. After incubation, the cells were washed with PBS and imaged over a 3-day period.

Gratifyingly, we only observed fluorescence with glycobrush-Chol treatment (Fig. 5b, c). No polymer fluorescence was observed in the case of treatment with glycobrush-N_3_, indicating that the Chol group is responsible for membrane insertion, and that the brushes are not nonspecifically endocytosed or adhered in these conditions. See the SI for comparison to control cells from a mock glycocalyx engineering experiment. Flow cytometry analysis indicated that glycocalyx engineering with our glycobrush-Chol structure is a robust and highly efficient process. Of the glycobrush-Chol-treated cells, 100% were AF594-positive as compared to only 1.5% of the glycobrush-N_3_-treated cells and only 0.3% of the mock-treated control (Fig. 5d and see SI). Chol-terminal glycobrushes were observed on the cell surface for up to 3 days with a half-life of 22 h (see SI).

We observed the brushes to be localized at both the cell surface and inside the cell, as indicated by the punctate spots. We conducted the same glycocalyx engineering experiment in conjunction with commercially available CF488A-transferrin, which is rapidly internalized into endosomes by invagination of clathrin-coated pits. As expected, we observed colocalization of the glycobrushes with transferrin (Fig. 5E–G). This indicates that, similar to other small molecule^53,54^, peptide^55,56^, and polymer^52^ Chol-conjugates, our brushes experience prolonged cell surface display due to endosomal recycling. Overall, these experiments indicate the potential of our materials to serve as components of a synthetic ECM or glycocalyx. Further studies are underway to determine how labeling efficiency and membrane residence time is affected by polymer composition.

In summary, we have developed a two-step one-pot method for the synthesis of glycosylated polypeptide bottlebrushes that mimic the structure of native glycoproteins. Our method enables complete tunability in terms of both backbone and branch MWs, branch graft density, and glycan identity and density. These materials are based entirely on amino acid and carbohydrate structures, and adopt native secondary structures. We found that monomers that yield helical glycopolypeptide branches polymerize with remarkably faster kinetics than those yielding rod-like or disordered structures. Analysis of the branches separately from the backbone revealed uniform chain growth even at 100% grafting density. The glycobrushes did not affect cell viability, are protease degradable, and, when functionalized with a cholesterol anchor, could be displayed on live cell surfaces. Overall, our method has solved long-standing challenges in the synthesis of biomimetic materials that encompass the high grafting density and dense glycosylation of native proteoglycans. Considering the growing body of evidence for the biological complexity and therapeutic applications of these fascinating biomolecules, there is a need for well-defined synthetic materials that harness their properties.

## Methods

Full experimental details, characterization of compounds, and additional data can be found in the SI.

### Polypeptide backbone synthesis

In an N_2_ atmosphere glove box, 6.4 mg of AMK NCA (0.0165 mmol) was dissolved in anhydrous THF. Ten microliters of a 30 mg/mL solution (0.826 μmol) of (PMe_3_)_4_Co in anhydrous THF was added to the NCA solution for a final NCA concentration of 50 mg/mL. The reaction was monitored by ATR-FTIR.

### Glycobrush synthesis

In an N_2_ atmosphere glove box, 2.16 mg of Ni(COD)_2_ (1 eq) was added to 51.4 μL of anhydrous THF. To the solution, 2.6 μL dmpe (2 eq.) was added and allowed to mix for 10 min. PAMK containing 7.85 μmol of activatable groups was dissolved in 131 μL of anhydrous DMF. The dmpeNi(COD) solution was added to the PAMK solution and the mixture was heated for 16 h at 80 °C. The activated backbone solution was cooled. NCAs used for the sidechain growth were dissolved at 50 mg/mL in anhydrous DMF. A volume of activated backbone solution was added to the NCA solution corresponding to the intended monomer : initiator ratio ([M]:[I]). Polymerizations were monitored by ATR-FTIR.

### Glycobranch cleavage

From the crude glycobrush polymerization reaction, a volume containing 2 mg of glycobrush was removed. To this solution, 0.5 mL of 40 mg/mL CNBr in 5 : 4 : 1 acetonitrile : acetic acid : water was added. The reaction was sealed and allowed to stand overnight. The reaction was evaporated to dryness. The solids were redissolved in 0.1 M LiBr in DMF at 3 mg/mL polymer and analyzed by GPC/LS.

### Cellular cytotoxicity assays

HEK293T cells were cultured in Delbucco’s modified Eagle medium (DMEM) with 10% fetal bovine serum (FBS), 2 mM l-glutamine, and 100 U/mL penicillin. Upon reaching sufficient confluency, cells were trypsinized and suspended in medium. Cells were loaded 5 × 10^3^ per well in a clear, flat-bottom 96-well plate coated with poly-l-Lys. Twenty-four hours after plating, cells were treated with 0.25 g/L polymer for 24 h, then analyzed using a CCK-8 assay from Dojingo Molecular Technologies, Inc. The CCK-8 reagent was allowed to incubate with cells for 3 h prior to absorbance reading.

### Glycocalyx engineering

AF594-labeled glycopolypeptides were dissolved at 15 µM in complete media (DMEM with 10% FBS, pen/strep, and l-glutamine) and sterile filtered through a 0.2 μm membrane. HEK293T cells were trypsinized and neutralized with complete media. The cells were pelleted by centrifugation at 100 × *g* for 5 m. Media was removed and then the cells were resuspended in media containing polymer. Cells were incubated in the media + polymer for 2 h at room temperature. Incubation could be conducted in the centrifuge tube, but transfer to a culture dish was preferred for improved surface area. Cells did not adhere at room temperature. Post incubation, treated cells were resuspended and centrifuged, washed with PBS, resuspended in complete media (lacking polymer), and plated. Untreated control cells were plated on a separate 24-well plate. All cells were left to grow at 37 °C. Cells were Hoescht stained and imaged under a fluorescent microscope (Laxco LMI-6000) at timepoints from 24 to 96 h following polymer treatment. See SI for studies with transferrin.

### Protease digestions

Next, 0.05% Gibco Trypsin was used at an E : S of 1 : 10 in a reaction buffer of 50 mM NH_4_HCO_3_ pH 8. METAP2 (from R&D Systems) was used at an E : S of 1 : 20 in a reaction buffer of 50 mM Hepes, 100 mM NaCl, 0.1 mM CoCl_2_ at pH 7.4. StcE was a gift from the lab of Carolyn Bertozzi. Protease K was obtained from ThermoFisher (#AM2542). Digestions with StcE and Proteinase K were performed in 1× PBS pH 7.4 with E : S of 1 : 10. All digestions were allowed to proceed for 48 h at 37 °C and with 40 μg of PAMK_63_-g-PGalNAcS_33_ per reaction at a concentration of 0.44 mg/mL. See SI for staining procedures.

### Reporting summary

Further information on research design is available in the Nature Research Reporting Summary linked to this article.

## Supplementary information

Supplementary Information

Reporting Summary

## Data Availability

All data needed to evaluate the conclusions in the paper are presented in the paper and/or the Supplementary Information. Raw data related to this paper are available from the corresponding author on reasonable request.
