# Supplementary material for: Tunable, biodegradable grafting-from glycopolypeptide bottlebrush polymers
Source: Nat Commun. 2021 Nov 9;12:6472. doi: 10.1038/s41467-021-26808-5 (PMC8578664; doi:10.1038/s41467-021-26808-5)
Supplement: Supplementary file 1 — Supplementary Information [file 41467_2021_26808_MOESM1_ESM.pdf]

Supplementary Information

Tunable, biodegradable grafting-from glycopolypeptide bottlebrush polymers

Zachary S. Clauss[1], Casia L. Wardzala[1], Austin E. Schlirf[1], Nathaniel S. Wright[1], Simranpreet S. Saini[1], Bibiana Onoa[2], Carlos Bustamante[2,3,4,5,6,7], Jessica R. Kramer[1,8]*

1. Department of Biomedical Engineering, University of Utah, Salt Lake City, Utah, 84102, USA. 2. Howard Hughes Medical Institute University of California Berkeley, Berkeley, CA 94720, USA. 3. Department of Chemistry, University of California Berkeley, Berkeley, CA 94720, USA. 4. Institute for Quantitative Biosciences, University of California, Berkeley, CA, 94720, USA. 5. Molecular Biophysics and Integrated Bioimaging Division, Lawrence Berkeley National Laboratory, Berkeley, CA 94720, USA. 6. Department of Physics, University of California Berkeley, Berkeley, CA 94720, USA. 7. Department of Molecular and Cell Biology, University of California Berkeley, Berkeley, CA 94720, USA. 8. Department of Pharmaceutics and Pharmaceutical Chemistry, University of Utah, Salt Lake City, Utah, 84102, USA. *e-mail: jessica.kramer@utah.edu

Table of Contents

Supplementary methods:

I. Instrumentation and General Methods **1**

II. Experimental Procedures **2**

II.a Chemical syntheses 2

II.b Polymerization and Polymer Modification Procedures 8

II.c Kinetic parameter determination 9

II.d AFM imaging and processing 10

II.e Protease digestion and gel staining protocols 10

II.f Glycocalyx engineering procedures 11

III. Supplementary Figures and Tables **12**

IV. NMR Spectra **23**

V. Supplementary References **27**

Supplementary Methods:

I. Instrumentation and General Methods

Reactions were conducted under an inert atmosphere of N_2, using oven-dried glassware unless otherwise stated. Hexanes and dichloromethane were purified by first purging with dry nitrogen, followed by passage through columns of activated 3Å molecular sieves. THF was purified by first purging with dry nitrogen,

followed by passage through columns of activated alumina. Anhydrous DMF was purchased from Sigma Aldrich and stored over 3Å molecular sieves. All oven dried glassware was dried at 120°C. Infrared spectra were recorded on a Bruker Alpha ATR FT-IR Spectrophotometer and data was analyzed with OPUS software package version 7.5. All polymerizations were monitored for completion via ATR FT-IR. Deionized water (18 MΩ-cm) was obtained by passing in-house deionized water through a Thermo Scientific MicroPure UV/UF purification unit. Tandem gel permeation chromatography/light scattering (GPC/LS) was performed on an Agilent 1260 Infinity liquid chromatograph pump equipped with a Wyatt DAWN HELEOS-II light scattering (LS) and a Wyatt Optilab T-rEX refractive index (RI) detectors and Astra 7.3.0 software package was used to analyze the data. Separations were achieved using 10^5, 10^4, and 10^3Å Phenomenex Phenogel 5 μm columns using 0.10 M LiBr in DMF as the eluent at 60 °C. All GPC/LS samples were prepared at concentrations of 3 mg/mL. ^{1}H NMR spectra were recorded on a Varian Mercury spectrometer (400 MHz) and are reported relative to deuterated solvent. Data for ^{1}H NMR are reported as follows: chemical shift (δ ppm), multiplicity, coupling constant (Hz) and integration. Data for ^{13}C NMR spectra are reported in chemical shift. Dynamic light scattering was performed on a Malvern Zetasizer at 1 g/L in plastic cuvettes. Canonical amino acid N-carboxyanhydrides were synthetized according to previous literature. CD measurements of the polypeptide solutions were recorded in quartz cells with a path length of 0.1 cm, on a JASCO J-1500 CD spectrophotometer. The mean residue ellipticity (MRE, [θ]) was calculated using the equation [θ]=(θ*m)/(c*l*n), where θ is measured ellipticity (mdeg), m is molecular weight (g/mol) of the glycopolypeptide, c is concentration (mg/mL), l is path length of the cuvette (cm), and n is the number of residues in the polypeptide. Dynamic light scattering (DLS) was performed on a Malvern Zetasizer Nano-ZS at a concentration of 1 mg/mL in MilliQ water. All samples were filtered through 0.22-micron filters immediately prior to the run.

II. Experimental Procedures

II.a Chemical syntheses

1,2,3,4,6-penta-O-acetyl-D-galactopyranoside (1a). Under N_2, a solution containing 75 mL of pyridine (74 g, 0.93 mol, 9.3 equiv) and 75 mL of acetic anhydride (81 g, 0.794 mol, 8.0 equiv) was cooled to 0°C. D-galactose (18.04 g, 0.1001 mol, 1.000 equiv) was added to the solution over 15 minutes. The reaction was stirred overnight and allowed to warm to room temperature. The reaction was transferred to a 1 L Erlenmeyer flask and cooled to 0°C. The solution was quenched with 300 mL of ice water added over 10 minutes. The resulting mixture was extracted with 4x160 mL ethyl acetate. The organic phases were combined and washed with 2x400 mL 1 M HCl, 3x100 mL sat. $NaHCO_3$, and 2x100 mL brine. The organic phase was dried over $MgSO_4$ and concentrated in vacou to yield 36.6 g of **1a** (93.4% yield) as an oil which was used without further purification.

2,3,4,6-tetra-O-acetyl-α-D-galactopyranosyl bromide (1b). Crude **1a** (29.6 g, 0.0758 mol, 1.0 equiv) was dissolved in 164 mL glacial acetic acid and cooled to 0°C under N_2. To the stirred solution, 55 mL of 33% HBr in acetic acid (24.5 g, 0.303 mol, 4.0 equiv) was added dropwise via an addition funnel. After addition was complete (~90 minutes), the solution was allowed to warm to room temperature and stirred overnight.

The reaction was quenched with 300 mL ice water. The reaction was diluted with 400 mL of ethyl acetate and transferred to a separatory funnel. The aqueous phase was extracted with 3x200 mL ethyl acetate. Combined organic phases were washed 5x400 mL sat. $NaHCO_3$, 2x150 mL brine, dried over Na_2SO_4 and concentrated in vacuo to yield 27.7 g of **1b** (88.8% yield). The ^{1}H-NMR spectrum agreed with literature.[1]

3,4,6-tri-O-acetyl-D-galactal (1c). Zn dust (43.37 g, 0.6632 mol, 9.000 equiv) were added to 220 mL of a 1:1 (v:v) mixture of acetic acid and water. The solution was cooled to 0°C under N_2. A 0.67 M solution containing **1b** in diethyl ether (30.3 g, 0.0737 mol, 1.00 equiv) was added dropwise over 1.5 hours via an addition funnel. The solution was allowed to warm to room temperature and stirred overnight. The reaction was transferred to a separatory funnel and extracted with 4x100 mL ethyl acetate. The organic phases were combined and washed with 5x200 mL sat. $NaHCO_3$ and 5x100 mL brine. The solution was dried over Na_2SO_4 and concentrated in vacuo. The crude product was purified in portions via flash chromatography with 33% ethyl acetate in hexanes. The ^{1}H-NMR spectra of the purified product agreed with literature.[1]

3,4,6-tri-O-acetyl-2-azido-2-desoxy-α-D-galactopyranosyl chloride (1d). **1c** (4.383 g, 0.01610 mol, 1.00 equiv) was dissolved in 100 mL of anhydrous acetonitrile (Alfa Aesar). The solution was cooled to -25°C and kept below -15°C for the entirety of the reaction. Iron (III) chloride hexahydrate (19.58 g, 0.07245 mol, 4.500 equiv) were added to the solution. After dissolution of the $FeCl_3$, sodium azide (3.14 g, 0.0483 mol, 3.00 equiv) and 1.49 mL of 30% H_2O_2 (0.493 g, 0.0483 mol, 3.0 equiv) were added in that order. The reaction remained below -15°C for 5 hours, when TLC indicated the consumption of **1c**. The reaction was concentrated to about 50 mL. The solution was diluted with ethyl acetate and washed with 5x50 mL water, 1x50 mL sat. $NaHCO_3$, and 1x50 mL brine. The solution was dried over Na_2SO_4 and concentrated in vacuo to yield a crude mass of 5.5 g (97% crude yield) which was used with no further purification. The ^{1}H-NMR spectrum agreed with literature.[2]

N-carbobenzyloxy-O-(3,4,6-tri-O-acetyl-2-azido-2-desoxy-α-D-galactopyranosyl)-L-serine benzyl ester (1e). Z-L-serine benzyl ester (2.21 g, 0.00678 mol, 0.566 equiv) was dried under vacuum overnight in the reaction flask prior to use. 33 mL of 1:1 (v:v) DCM:toluene, 7.8 g 3Å molecular sieves, and Ag_2CO_3 (2.80 g, 0.0102 mol, 0.849 equiv) were added to the reaction vessel under N_2. The reaction was cooled to 0°C. $AgClO_4$ (0.363 g, 0.00169 mol, 0.142 equiv) was added and the reaction was covered in aluminum foil. **1d** (4.2 g, 0.012 mol, 1.0 equiv) was dissolved in 33 mL DCM and added to the reaction over 15 minutes. The reaction was allowed to warm to room temperature and stirred overnight. The reaction was filtered over Celite, diluted with 65 mL ethyl acetate and washed with 1x100 mL water, 1x100 mL sat. $NaHCO_3$, and 2x100 mL brine. The solution was dried over Na_2SO_4 and concentrated in vacuo. The crude yield was 5.14 g, which used without further purification. The ^{1}H-NMR spectrum agreed with literature**.**[3]

N-carbobenzyloxy-O-(3,4,6-tri-O-acetyl-2-acetamido-2-deoxy-α-D-galactopyranosyl)-L-serine benzyl ester (1f). The crude product (5.14 g) was dissolved in 228 mL of a mixture of THF:acetic anhydride:acetic acid (3:2:1, v:v:v). Zn dust (6.28 g, 0.09587 mol, 12.0 equiv) and 30 mL of saturated $CuSO_4$ were added. After 50 minutes, the reaction was determined to be complete according to TLC (33% ethyl acetate in hexanes with p-anisaldehyde stain). The reaction was filtered over Celite and concentrated.

The concentrate was redissolved in ethyl acetate, washed with 3x50 mL water, 5x100 mL sat. $NaHCO_3$, and 2x100 mL brine. The solution was dried over Na_2SO_4 and concentrated in vacuo. The crude mass was 4.4 g. The product was purified via flash chromatography (2:1 ethyl acetate:hexanes). The purification yielded 1.9 g of α anomer from 3.1 g of crude. ^{1}H-NMR (400 MHz, $CDCl_3$, 25 °C): δ 7.36 (m, 10 H), 5.13 (m, 4H), 5.02 (dd, 1H, 11.3, 3.2 Hz), 4.77 (d, 1H, 3.7 Hz), 4.60 (m, 1H), 4.52 (m, 1H), 4.12 (q, 1H, 7.1 Hz), 4.08-3.88 (m, 4), 2.14 (s, 3H), 2.04 (s, 3H), 2.00 (s, 3H), 1.90 (s, 3H).

O-(3,4,6-tri-O-acetyl-2-acetamido-2-deoxy-α-D-galactopyranosyl)-L-serine (1g). 0.38 g Pd/C was suspended in 100 mL MeOH under N_2. **1f** (1.9 g, 0.0029 mol, 1.0 equiv) was dissolved in 13 mL MeOH and added slowly to the Pd/C solution. The reaction was stirred under an H_2 atmosphere and was monitored via TLC. The reaction was complete in 3 hours. The reaction was filtered and the filtrate was concentrated in vacuo. The crude reaction product was redissolved in minimal MeOH and precipitated into 10x volume of 1:1 hexanes:ether. The precipitation was repeated two times. ^{1}H-NMR (400 MHz, CD_3OD, 25 °C): δ 5.41 (d, 1H, 1.6 Hz), 5.20 (dd, 1H, 11, 3.3 Hz), 4.91 (d, 1H, 3.3 Hz), 4.50 (dd, 1H, 11.5, 4.1 Hz), 4.30 (t, 1H, 6.8 Hz), 4.17 (m, 1H), 4.09 (m, 2H), 3.83 (m, 1H), 3.78 (br s, 1H), 2.14 (s, 3H), 2.03 (s, 3H), 1.97 (s, 3H), 1.94 (s, 3H).

O-(3,4,6-tri-O-acetyl-2-acetamido-2-deoxy-α-D-galactopyranosyl)-L-serine N-carboxyanhydride (1h). To a solution containing **1g** (1.07 g, 0.00246 mol, 1.0 equiv) dissolved in 55 mL anhydrous THF under N_2, α-pinene (1.34 g, 0.00983 mol, 5.0 equiv) and 3.5 mL of a 15 wt% phosgene (81 g, 0.00492 mol, 2.0 equiv) in toluene solution were added. *Caution: Phosgene is extremely hazardous and all manipulations must be performed in a well-ventilated chemical fume hood with proper personal protection and necessary precautions taken to avoid exposure.* The reaction was stirred overnight under static N_2 and confirmed completed by ATR FT-IR. The mixture was concentrated under high vacuum for 1 h using two sequential liquid nitrogen-cooled traps to condense solvent, byproducts, and phosgene. The collected condensate was quenched with ammonium hydroxide to consume residual phosgene prior to disposal. The crude NCA was dissolved in minimal anhydrous dichloromethane and purified by flash chromatography on anhydrous silica gel (5-20% THF in dichloromethane). The ^{1}H-NMR spectrum agreed with literature.[3]

1-allyl-2,3,4,6-tetra-O-acetyl-α,β-D-glucopyranoside (2a). Penta-O-acetyl-D-glucopyranoside (10.06 g, 0.02577 mol, 1.0 equiv) was dissolved in 53 mL of dry acetonitrile under N_2. The solution was cooled to 0°C and 12.3 mL of allyltrimethylsilane (8.83 g, 0.0773 mol, 3.0 equiv) was added. Boron trifluoride etherate (18.29 g, 0.1289 mol, 5.0 equiv) was then added drop wise over 15 minutes. The reaction was stirred at 0°C for 2 hours and then allowed to warm to room temperature overnight. The reaction was slowly poured into 200 mL cold sat. $NaHCO_3$ and stirred until the evolution of gases ceased. The mixture was extracted with 3x100 mL dichloromethane, washed with brine, and dried over Na_2SO_4. The solution was concentrated under vacuum and purified via flash chromatography (2:1 hexanes:ethyl acetate) to yield 1.69 g of pure α anomer and 0.32 g of mixed fractions. Low yield was attributed to the quality of allyltrimethylsilane. Mixed franctions were recrystallized from 2:1 hexanes:ethyl acetate to yield another 100 mg. The ^{1}H-NMR spectrum matched literature.[4]

2,3,4,6-tetra-O-acetyl-α-D-glucopyranosyl acid (2b). 2a (1.39 g, 0.00373 mol, 1.00 equiv) was dissolved in 22 mL of water:MeCN:ethyl acetate (3:2:2). $RuCl_3$ (0.043 g, 0.00015 mol, 0.040 equiv) was added followed by the addition of $NaIO_4$ (6.547 g, 0.03061 mol, 8.200 equiv). The reaction was monitored via TLC and determined complete within two hours. The solution was diluted with 50 mL water and extracted with 4x50 mL dichloromethane. The organic layers were combined and washed 1x20 mL brine. The solution was concentrated to yield 1.12 g of **2b** (76.7% yield). The ^{1}H-NMR spectrum matched literature.[4]

N-hydroxysuccinimide ester of 2,3,4,6-tetra-O-acetyl-α-D-glucopyranosyl acid (2c). 5 mL of anhydrous THF was added to **2b** (0.335 g, 0.00086 mol, 1.00 equiv) and N-hydroxysuccinimide (0.119 g, 0.00103 mol, 1.20 equiv) in a flask under N_2. The solution was cooled to 0°C. DCC (0.186 g, 0.00090 mol, 1.05 equiv) was added. The solution formed a white precipitate after about 5 minutes. At this point, the ice bath was removed, and the reaction was allowed to stir at room temperature for 1 hour. Then, the reaction flask was sealed with a stopper and placed in a 4°C fridge overnight. The precipitated DCU was filtered off, the filtrate concentrated, and the concentrate redissolved in dichloromethane. The flask was placed in the fridge overnight at 4°C to precipitate more DCU. DCU was removed by filtration, and the filtrate was concentrated to yield 0.26 g (62% yield). The ^{1}H-NMR spectrum matched literature.[4]

N-carbobenzyloxy-L-lysine-1-(2,3,4,6-tetra-O-acetyl-α-D-glucopyranoside) (2d). To a solution containing **2c** (1.394 g, 0.00286 mol, 1.0 equiv), α-Z-L-lysine (1.203 g, 0.00429 mol, 1.500 equiv) and 0.384 g of $NaHCO_3$ (0.384 g, 0.00458 mol, 1.60 equiv) were added. 4 mL of water was added to solubilize $NaHCO_3$. The reaction was stirred for 3 hours at room temperature and then concentrated to about half the starting volume in vacuo. The reaction was acidified to pH 2 with 1 M HCl and extracted with 3x50 mL dichloromethane. The crude was purified via flash chromatography with an eluent of 2 parts methanol, 1 part acetic acid, and 100 parts ethyl acetate to yield 1.7 g of **2d** (91% yield)**.** The ^{1}H-NMR spectrum matched literature.[4]

L-lysine-1-(2,3,4,6-tetra-O-acetyl-α-D-glucopyranoside) N-carboxyanhydride (AMK NCA) (2e). To a solution of of **2d** (0.81 g, 0.00124 mol, 1.0 equiv) in 43 mL of anhydrous dichloromethane under N_2, α,α-dichloromethylmethyl ether (0.357 g, 0.00310 mol, 2.5 equiv) and α-pinene (0.845 g, 0.00621 mol, 5.0 equiv) were added. An oven dried reflux condenser was added and the reaction was stirred for 36 hours at 45°C. The reaction was determined complete upon ATR FT-IR confirmation of the characteristic N-carboxyanhydride carbonyl stretches at 1852 and 1782 cm^{-1}. The reaction was condensed. The crude NCA was dissolved in minimal anhydrous dichloromethane and purified by flash chromatography on anhydrous silica gel[4] with an eluent gradient of two column volumes of 40%, THF in hexanes, two column volumes of 60% THF in hexanes, and four column volumes of 80% THF in hexanes. In total, 23 fractions were collected, and fractions 13-17 were combined since NCA was detected via ATR FT-IR. The column yielded 450 mg of **2e** (66% yield).

octa-O-acetyl-D-lactose (3a). To a 0°C stirred solution of 22 mL of pyridine (22 g, 0.27 mol, 20. equiv) and 22 mL of acetic anhydride (24 g, 0.23 mol, 17.0 equiv), lactose monohydrate (5.000 g, 0.0139 mol, 1.000 equiv) was added over 10 minutes. The reaction was allowed to warm to room temperature and stirred

overnight. The reaction was cooled to 0°C and quenched with 100 mL of water. The reaction was extracted with 3x50 mL ethyl acetate. The organic layers were combined and washed with 3x80 mL 1 M HCl, 4x90 sat. $NaHCO_3$, and 3x80 mL brine. The solution was dried over Na_2SO_4 and concentrated in vacuo to yield 8.4 g of **3a** (89% yield) which was used without further purification. ^{1}H-NMR (400 MHz, $CDCl_3$, 25 °C): δ 6.25 (d, 1 H, 3.6 Hz), 5.46 (t, 1H, 9.8 Hz), 5.36 (d, 1H, 3.0 Hz), 5.12 (dd, 1H, 10.4, 7.9 Hz), 5.01 (dd, 1H, 10.1, 3.6 Hz), 4.96 (dd, 1H, 10.4, 3.4 Hz), 4.48 (d, 1H, 7.9 Hz), 4.45 (dd, 1H, 12, 1.5 Hz), 4.18-4.06 (m, 4), 4.00 (m, 1H), 3.88 (t, 1H, 7 Hz), 3.81 (t, 1H, 9.6 Hz), 2.18 (s, 3H), 2.16 (s, 3H), 2.13 (s, 3H), 2.06 (s, 3H), 2.06 (s, 3H), 2.01 (s, 3H), 1.97 (s, 3H).

1-bromo-hepta-O-acetyl-α-D-lactose (3b). A stirred solution containing **3a** (8.4 g, 0.012 mol, 1.0 equiv) in 120 mL anhydrous dichloromethane was cooled to 0°C under N_2. 9.0 mL of a 33% HBr in AcOH (4.0 g, 0.050 mol, 4.0 equiv) solution was added dropwise over 45 minutes. Then, the reaction was allowed to warm to room temperature and stirred overnight. The reaction was cooled to 0°C and slowly quenched with 100 mL of ice water. The mixture was transferred to a separatory funnel. The aqueous layer was extracted 1x20 mL dichloromethane. The organic layers were combined and washed with 2x100 mL sat. $NaHCO_3$ and 1x50 mL brine. The organic layer was dried over Na_2SO_4 and concentrated in vacuo. The crude product 7.5 g (86% yield) was used without further purification.

N-carbobenzyloxy-O-(hepta-O-acetyl-β-D-lactose)-L-serine benzyl ester (3c). Z-L-serine benzyl ester (0.724 g, 0.00220 mol, 0.769 equiv) was dried under vacuum overnight in the reaction flask prior to use. 39 mL of anhydrous dichloromethane, 4.6 g 3Å molecular sieves, and **3b** (2.00 g, 0.00286 mol, 1.00 equiv) were added to the reaction vessel under N_2**.** The reaction was cooled to 0°C and covered with aluminum foil. AgOTf (0.801 g, 0.308 mol, 1.08 equiv) was added over 30 minutes in 3 portions. The reaction was allowed to warm to room temperature and stirred overnight. The reaction was cooled to 0°C and quenched with 0.46 mL triethylamine over 15 minutes. The reaction was filtered over Celite. The filtrated was washed with 1x100 mL sat. $Na_2S_2O_3$, 1x100 mL $NaHCO_3$, and dried over Na_2SO_4. The residue was purified via flash chromatography using 33%-75% ethyl acetate in hexanes to yield 0.683 g of **3c** β anomer (34% yield). The ^{1}H-NMR spectrum agreed with literature.[5]

O-(hepta-O-acetyl-β-D-lactose)-L-serine (3d). 0.100 g Pd/C was added to a flask filled with N_2. 0.500 g of **3c** (0.500 g, 0.00054 mol, 1.0 equiv) was dissolved in 21 mL MeOH and added slowly to the reaction vessel. The reaction was stirred under an H_2 atmosphere. The reaction was monitored via TLC and determined complete within 3 hours. The reaction was filtered and the filtrate was concentrated in vacuo. The crude reaction product was redissolved in minimal MeOH and precipitated into 10x volume of 1:1 hexanes:ether. The precipitation was repeated three times to yield 0.320 g of **3d** (82% yield). ^{1}H-NMR (400 MHz, CD_3OD, 25 °C): δ 5.35 (d, 1 H, 3.4 Hz), 5.19 (t, 1H, 9.2 Hz), 5.15-4.96 (m, 2H), 4.88 (dd, 1H, 9.5, 8.6 Hz), 4.71 (dd, 2H, 10.9, 8.3 Hz), 4.61 (d, 1H, 12.6 Hz), 4.18-4.08 (m, 5H), 4.00 (t, 1H, 9.9 Hz), 3.88 (t, 1H, 9.5 Hz), 3.80-3.74 (m, 1H), 3.71 (dd, 1H, 8.8, 3.2 Hz), 2.13 (s, 3H), 2.11 (s, 3H), 2.05 (s, 3H), 2.03 (s, 3H), 2.03 (s, 3H), 2.03 (s, 3H), 1.92 (s, 3H).

O-(hepta-O-acetyl-β-D-lactose)-L-serine N-carboxyanhydride (3e). 3d (0.540 g, 0.000746 mol, 1.0 equiv) was dissolved in 16 mL anhydrous THF under N_2. 0.474 mL α-pinene (0.407 g, 0.00298 mol, 4.0 equiv) was added. 1.1 mL of a 15wt% phosgene solution in toluene (0.148 g, 0.00149 mol, 2.0 equiv) was added and the reaction vessel was filled with N_2 and sealed. *Caution: Phosgene is extremely hazardous and all manipulations must be performed in a well-ventilated chemical fume hood with proper personal protection and necessary precautions taken to avoid exposure.* The reaction was stirred for 48 hours under and determined to be completed by ATR FT-IR. The mixture was concentrated under high vacuum and the condensate was quenched with ammonium hydroxide. The crude NCA was dissolved in minimal anhydrous dichloromethane and purified by flash chromatography on anhydrous silica gel (50-70% THF in hexanes) to afford 0.435 g (78% yield). The ^{1}H-NMR spectrum agreed with literature.[6]

L-glutamic acid γ-benzyl ester N-carboxyanhydride (4) was synthesized according to previous literature.[7]

$N_ε$-carbobenzyloxy-L-lysine N-carboxyanhydride (5) was synthesized according to previous literature.[8]

3-amino-5-cholestene (6). 3-amino-5-cholestene was synthesized according to literature and ^{1}H-NMR spectra matched those previously published.[9]

N-allyloxycarbonyl-L-isoleucine (7) was synthesized according to previous literature.[3]

N-allyloxycarbonyl-L-isoleucine-amido-5-cholestene (8). 7 (0.155 g, 0.72 mmol, 1.0 equiv) was dissolved in 2 mL DMF and stirred. **6** (0.279 g, 0.72 mmol, 1.0 equiv) was dissolved in 5 mL of a 2:3 DMF:THF mixture and added to the first solution. The resulting solution was hazy. (2-(1H-benzotriazol-1-yl)-1,1,3,3-tetramethyluronium hexafluorophosphate (0.274 g, 0.72 mmol, 1 equiv) was added to the reaction mixture, and the solution cleared up. Triethylamine (TEA) (0.146 g, 1.45 mmol, 2 equiv) was added dropwise to the solution. The reaction become slightly cloudy during addition of TEA, which again cleared after addition was completed. The reaction was allowed to stir overnight. The reaction was concentrated to remove THF and then diluted with DCM. The organic layer was washed 3x10 mL 1 M HCl, 3x10 mL saturated sodium bicarbonate, 1x20 mL brine, and then dried over sodium sulfate. The crude had a mass of 0.492 g. The crude was purified via flash chromatography with an eluent of 17.5% ethyl acetate in hexanes to afford a mass of 305 mg (72% yield). ^{1}H-NMR (400 MHz, $CDCl_3$, 25°C): δ 5.96-5.86 (m, 1H), δ 5.78 (d, 1H, 8.4 Hz), δ 5.36 (s, 1H), δ 5.30 (t, 2H, 8.5 Hz), δ 5.22 (d, 1H, 10.7 Hz), δ 5.14 (d, 1H, 7.5 Hz),), δ 4.56 (d, 2H, 4.7 Hz),), δ 4.08 (br m, 1H),), δ 3.67 (br m, 1H),), δ 2.28 (d, 1H, 12.7 Hz),), δ 2.15-1.90 (br m, 3H), δ 1.88-1.75 (br m, 3H), δ 1.71-0.84 (br m, 41H), δ 0.67 (s, 3H). ^{13}C-NMR (125 MHz, $CDCl_3$, 25°C): δ 171.2, δ 156.1, δ 140.0, δ 132.6, δ 122.1, δ 117.8, δ 65.8, δ 56.7, δ 56.1, δ 53.6, δ 50.1, δ 49.8, δ 42.3, δ 41.7, δ 39.7, δ 39.5, δ 39.2, δ 37.8, δ 36.5, δ 36.2, δ 35.8, δ 31.8, δ 28.9, δ 28.2, δ 28.0, δ 24.7, δ 24.3, δ 23.8, δ 22.9, δ 22.8, δ 22.5, δ 22.1, δ 20.9, δ 19.3, δ 18.7, δ 11.8.

Nε-(allyloxycarbonyl-L-methionyl)-L-lysine-N-carboxyanhydride (9) and Nε-(allyloxycarbonyl-L-isoleucyl)-L-lysine-N-carboxyanhydride (10) were both synthesized according to literature and ^{1}H-NMR spectra matched those previously published.[7]

Methoxy polyethylene glycol isocyanate (PEG-NCO). Methoxy-PEG-amine 1 kDa (0.36 g, 0.00036 mol, 1.0 equiv) were dissolved in 18 mL of anhydrous THF. 0.514 mL of a 15 wt% phosgene solution in toluene (0.071 g, 0.00072 mol, 2.0 equiv) was added to the solution. *Caution: Phosgene is extremely hazardous and all manipulations must be performed in a well-ventilated chemical fume hood with proper personal protection and necessary precautions taken to avoid exposure.* The reaction was stirred for 18 hours at room temperature under N_2. The mixture was concentrated under high vacuum and the collected condensate was quenched with ammonium hydroxide. The crude PEG-NCO was precipitated from minimal THF into 1:1 ether:hexanes three times to yield 280 mg (77% yield).

II.b Polymerization and Polymer Modification Procedures

General procedure for polymerization using Co[0] catalyst. 6.4 mg of AMK NCA (0.0165 mmol) was dissolved in anhydrous THF inside a N_2 atmosphere glove box. 10 µL of a 30 mg/mL solution (0.826 µmol) of $(PMe_3)_4$ Coin anhydrous THF was added to the NCA solution for a final NCA concentration of 50 mg/mL. The reaction was complete within a few hours via ATR FT-IR.

General procedure for backbone activation with Ni[0] catalyst. 2.16 mg of $Ni(COD)_2$ (1 eq) was added to 51.4 µL of anhydrous THF inside a N_2 atmosphere glove box. To the solution, 2.6 µL dmpe (2 eq.) was added. Over 10 minutes, the solid $Ni(COD)_2$ reacted with the dmpe and dissolved to form NiCODdmpe. 7.85 µmol of activatable groups on PAMK from the above polymerization was transferred to a thick-walled glass tube. 131 µL of anhydrous DMF was added to the tube followed by the NiCODdmpe solution. The tube was sealed with a Teflon stopper and transferred to an oil bath outside of the glove box for 16 hours at 80°C. The tube was cooled and transferred back into the glove box for further use.

General procedure for brush side chain growth. Inside a N_2 atmosphere glove box, the activated backbone solution volume was measured via syringe to determine the concentration. The NCA used for the side chain growth was dissolved in anhydrous DMF (final concentration of 50 mg/mL). A known volume of activated backbone solution was added to the NCA solution corresponding to the intended monomer:initiator ratio ([M]:[I]). Most polymerizations were left overnight and determined complete by ATR FT-IR.

General procedure for branch cleavage. From the crude glycobrush polymerization reaction, a volume containing 2mg of glycobrush was removed. To this solution, 0.5 mL of 40 mg/mL cyanogen bromide in 5:4:1 acetonitrile:acetic acid:water was added. The reaction was sealed and allowed to stand overnight. The reaction was evaporated to dryness. The solids were redissolved in 0.1 M LiBr in DMF at 3 mg/mL polymer and analyzed by GPC/LS.

General procedure for tert-butyl glutamate deprotection. Postpolymerization, the glycopolypeptide was transferred into a 4 mL vial and concentrated in vacuo. Enough trifluoroacetic acid (TFA) was added to the resulting oil to cover it and stirred for 30 minutes. TFA was then removed in vacuo.

General procedure for acetate deprotection. The glycopolypeptide was transferred to a 4 mL vial and concentrated in vacuo. 0.25 M K_2CO_3 in 1:1 MeOH:H_2O was added and all polymer solids were immersed

in the solution. The suspension was stirred overnight and polymer solids had dissolved after ca 16 hours. The solution was transferred to 1 kDa cut-off dialysis bags and dialyzed against deionized water for three days changing the water twice per day. The solution was lyophilized to afford a white foam.

General procedure for PEG endcapping for molecular weight determination. To a solution of polypeptide, 5 equivalents of PEG-NCO per mole of catalyst used in the polymerization reaction was added. The reaction was allowed to stand overnight. Endcapped polypeptides were precipitated from minimal THF into water three times to remove excess PEG. Then dried under vacuum or lyophilized. If the polymer was glycosylated, the polymer was then deprotected and dialyzed as described above. The ^{1}H-NMR integration of the ethylene protons on PEG were calibrated to 88 protons and the integral ratio of polypeptide protons was used for M_n determination.

General procedure for AF594 conjugation to glycobrushes. Glycobrush (5-7 mg) was dissolved in about 400 µL MilliQ water. A solution of 1 M $NaHCO_3$ (40 uL) was added. A solution of fluorophore (AF594-NHS, 5 g/L, 8 molar equiv. per brush) was added and the reaction allowed to stand for 24 hours. Excess fluorophore was removed using 3 KDa cut-off spin filters. The dye conjugation efficiency was calculated by obtaining an absorbance spectrum of the purified, dye labeled brush solution. Using the known Alexa Fluor extinction coefficient, 92,000 cm^{-1} M^{-1} at the emission maximum 617nm, we calculated 0.28 and 0.34 dye molecules per N_3- and Chol-labeled brush molecule, respectively.

II.c Kinetic parameter determination

Kinetic parameter analysis. Deconvolution of ATR FT-IR spectra was conducted using Matlab 2016a by Non-Linear Least-Squares Minimization and Curve-Fitting for Python 3.7.[10] Voight distributions were used for all carbonyl absorption peaks. The area under the carbonyl absorption curve, *A*, was used for each calculation. R(t) represents the ratio of the areas under larger anhydride carbonyl absorption peak, A_{1790}, and the area under the acetyl carbonyl absorption peak, A_{1740}.

$$R(t) = \frac{A_{1790}(t)}{A_{1740}(t)}$$

The concentration of monomer at any time is represented by the following equation.

$$[M] = [M]_0 \left(\frac{R(t)}{R(t_0)}\right)$$

Where $R(t_0)$ is the ratio obtained in a pure NCA thin film, without polymerization.

Second-order kinetic parameters were obtained from linear regression from ln[M] versus time plots through a pseudo first-order analysis[11] since the catalyst concentration remains constant as catalyst directly translate to the number of active growing chains, and second order kinetic parameters were found based on the equations below.

$$[M] = [M_0]e^{-k_{obs}t}$$

$$\frac{d[M]}{dt} = -k_{obs}[M]$$

$$k_{obs} = k_2[catalyst]$$

II.d AFM imaging and processing

Atomic Force Microscopy (AFM) sample preparation and imaging:
Solutions of glycobrush in Milli-Q water (10 µL of 10-20 nM) were deposited on freshly cleaved bare mica for 1 minute, rinsed with Milli-Q water, then gently dried under a stream of N2 perpendicular to the mica surface. AFM measurements were taken with a MultiMode NanoScope V atomic force microscope (Bruker Co.) equipped with a vertical engagement scanner E. The samples were imaged in tapping mode using silicon cantilevers (Nanosensors), excited at their resonance frequency (280-350 kHz) with free amplitudes (Ao) of 2-10 nm. The image amplitude (set point As) and A0 ratio (As/A0) was kept at ~0.8 in a repulsive tip-sample interaction regime, and phase oscillations no greater than ±5 degrees. The surface was rastered following the fast scan axis (x) at rates of 1.5- 2 Hz, the retrace line was captured to reconstruct the AFM micrographs. All samples were measured at room temperature in air, at a relative humidity of 30%.

AFM image processing and masking. Images were processed with Gwyddion version 2.53. Only molecules that were equilibrated on the mica surface and fully contained within images were considered. Grains were masked in a multi-step process as follows. First, a zero height was set as the zero mean value. Then, grains were masked by a height threshold of 30.5%. Lastly, grains were filtered by a minimum area of 28 pixels and a height limit between 0.46 nm and 1.2 nm. These masking parameters allow for removal of multiple polymer chains aggregated together as well as background signals. The volume of each grain was determined by the zero-basis volume. RStudio v3.6.1 was used to create the violin plot.

II.e Protease digestion and gel staining protocols

Protease digestions. 0.05% Gibco Trypsin was used at an E:S of 1:10 in a reaction buffer of 50 mM NH_4HCO_3 pH 8. Methionine aminopeptidase 2 (METAP2 from R&D Systems) was used at an E:S of 1:20 in a reaction buffer of 50 mM Hepes, 100 mM NaCl, 0.1 mM $CoCl_2$ at pH 7.4. StcE was a gift from the lab of Carolyn Bertozzi and was expressed and purified according to literature.[12] Protease K was obtained from ThermoFisher (#AM2542). Digestions with StcE and Proteinase K were performed in 1X PBS pH 7.4 with

E:S of 1:10. All digestions were allowed to proceed for 48 hours at 37°C and with 40 μg of $PAMK_{63}$-*g*-$PGalNAcS_{33}$ per reaction at a concentration of 0.44 mg/mL.

Electrophoresis. Bis-tris 4-12% gels from BioRad were used. Digestions were diluted with 4X Loading Buffer from BioRad. 10 ug of glycobrush digestion were loaded into each lane. Gels ran at 175V for 40 minutes. To visualize the glycobrush, Pro-Q ® Emerald 300 Lipopolysaccharide Gel Stain Kit from ThermoFisher was used. Gels were imaged on a standard gel imager.

II.f Glycocalyx engineering procedures

AF594-labeled glycopolypeptides were dissolved at 15 μM in complete media (DMEM with 10% FBS, pen/strep, and L-glutamine) and sterile-filtered through a 0.2 μm membrane. HEK293T cells were trypsinized and neutralized with complete media. Cells were pelleted by centrifugation at 200xg for 5 minutes. Media was removed and cells were resuspended in media containing polymer. Cells were then incubated in the media + polymer for 2 hours at room temperature. Incubation could be conducted in the microcentrifuge tube, but transfer to a culture plate was preferred for improved surface area. Cells did not adhere at room temperature. Post incubation, treated cells were resuspended and centrifuged, washed with PBS, resuspended in complete media (lacking polymer), and plated. Mock-engineered control cells were plated on a separate 24-well plate. All cells were left to grow at 37 °C. At timepoints from 24-96 hours following polymer treatment, cells were Hoescht 33342 stained and imaged under a fluorescent microscope (Laxco LMI-6000). Fluorescence was measured with ImageJ version 1.51j8 via fluorescence thresholding and pixel quantification. AF594 fluorescence was normalized to the Hoescht fluorescence and plotted against time. An exponential decay fit was applied in GraphPad version 6 and used to estimate the half-life. Minimal fluorescence from the glycobrushes was observed at 96 hours, however Hoescht stain toxicity affected cellular viability over this prolonged period. Therefore, we utilized data up to 72 hours for these calculations.

II.h Flow cytometry

Cells were coated with AF594-labeled glycopolypeptides according to the above protocol. After the PBS wash outlined above, mock engineered and treated cells were resuspended in PBS at ~10^6 cells/mL. DAPI was added as a live/dead discriminator. Data was acquired on a Beckman Coulter Cytoflex S flow cytometer and analyzed using Flow Jo v10.8.0. The gating tree was as follows: 1) FSC/SSC to 2) live gate (DAPI negative) to 3) SSC/PE (AF594 positive). See SI Fig S21 for gating.

II.g Transferrin colocalization study

Cells were coated with AF594-labeled glycopolypetides according to above protocol in serum-free media. CF488A-transferrin was added to a final concentration of 30 μg/mL for the last 30 minutes of a 2-hour incubation with polymer. Media was exchanged for complete media, and cells were incubated at 37°C for 15 minutes to allow for transferrin trafficking. Cells were washed with PBS, fixed with 4% paraformaldehyde, and fluorescently imaged.

III. Supplementary Figures and Tables

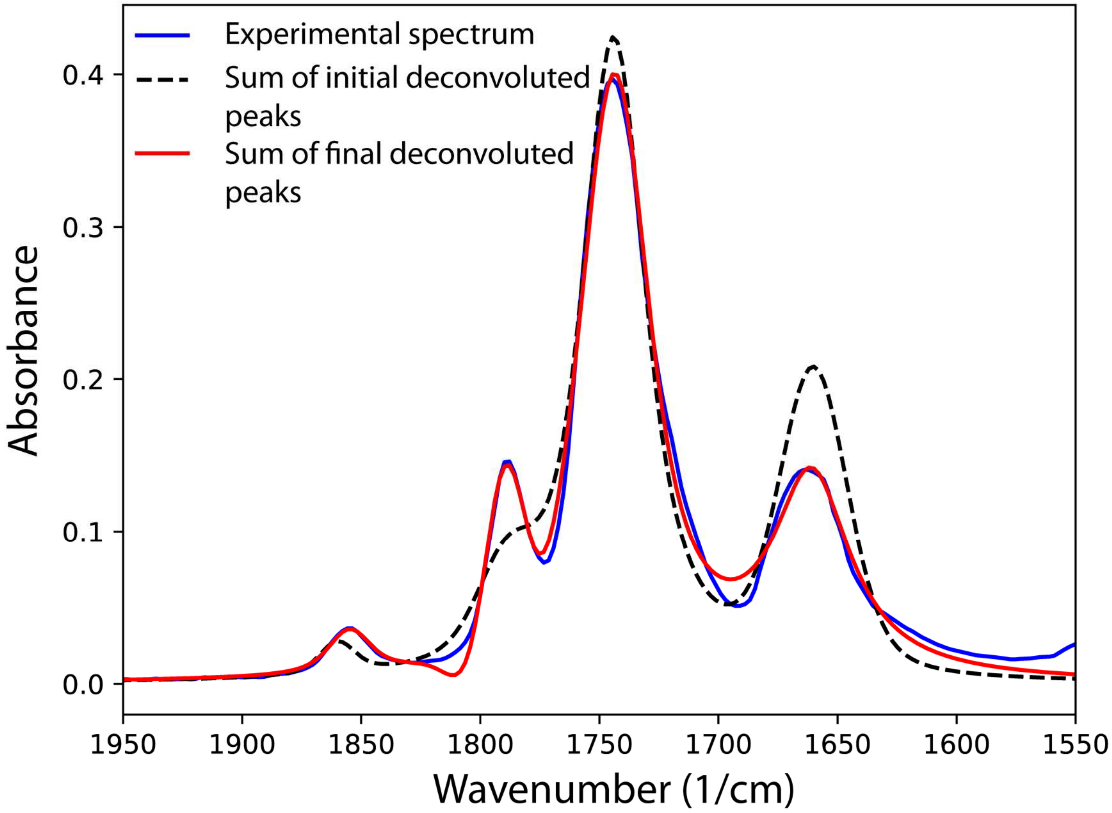

Supplementary Figure 1. Representative sum of deconvoluted peaks of ATR-FTIR spectrum using LM-FIT for Python for kinetic parameter determination. 3-hour time point from $(PAMK_{0.25}$-*s*-$PZK_{0.75})_{75}$-*g*-$PLacS_{25}$.

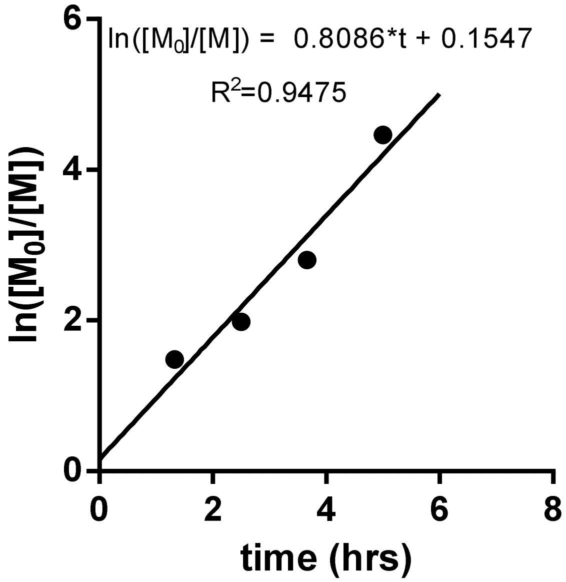

Supplementary Figure 2. First-order kinetic fit of deconvolution data of $PAMK_{63}$-*g*-$PGlcK_{117}$.

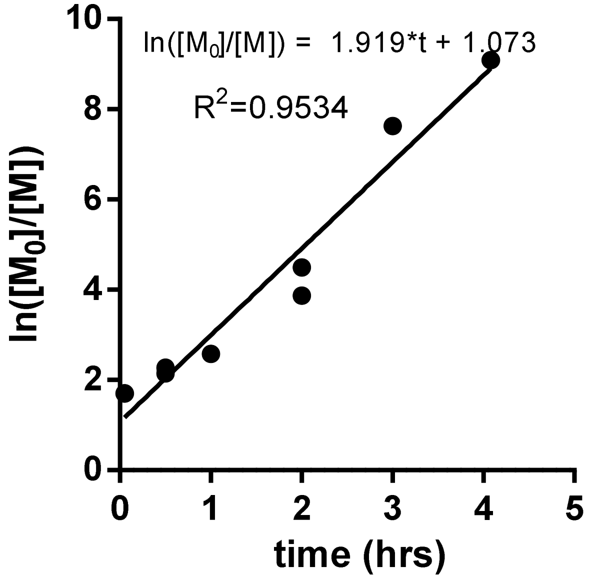

Supplementary Figure 3. First-order kinetic fit of deconvolution data of $(PAMK_{0.25}$-s-$PZK_{0.75})_{75}$-g-$PGlcK_{50}$.

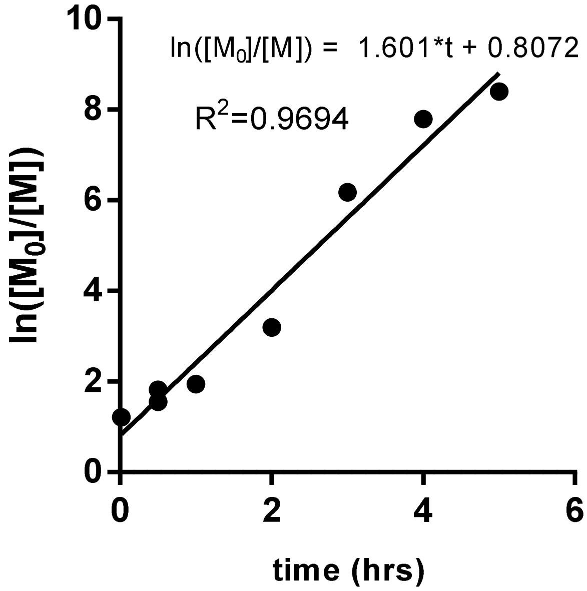

Supplementary Figure 4. First-order kinetic fit of deconvolution data of $(PAMK_{0.25}\text{-s-}PZK_{0.75})_{75}\text{-g-}PGlcK_{68}$.

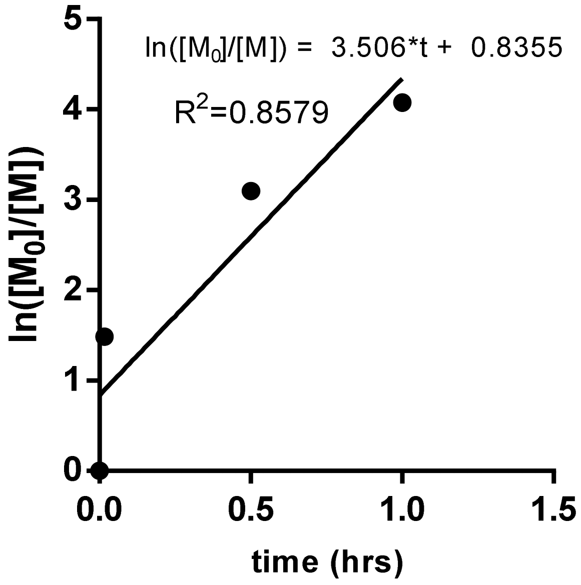

Supplementary Figure 5. First-order kinetic fit of deconvolution data of $PGlcK_{99}$.

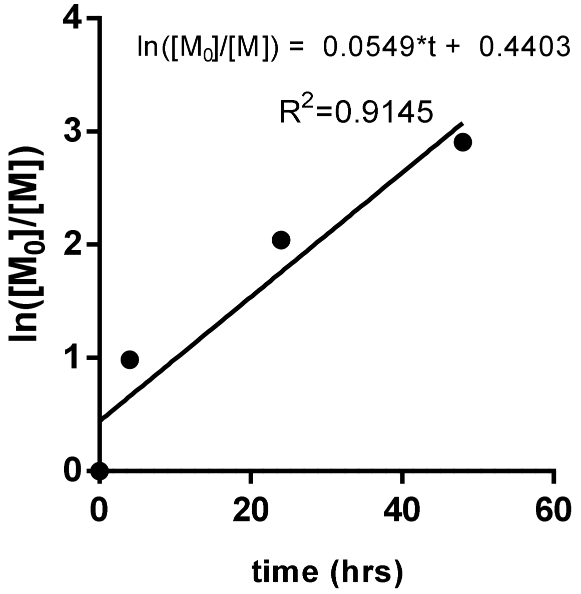

Supplementary Figure 6. First-order kinetic fit of deconvolution data of PAMK$_{63}$-*g*-PGalNAcS$_{33}$.

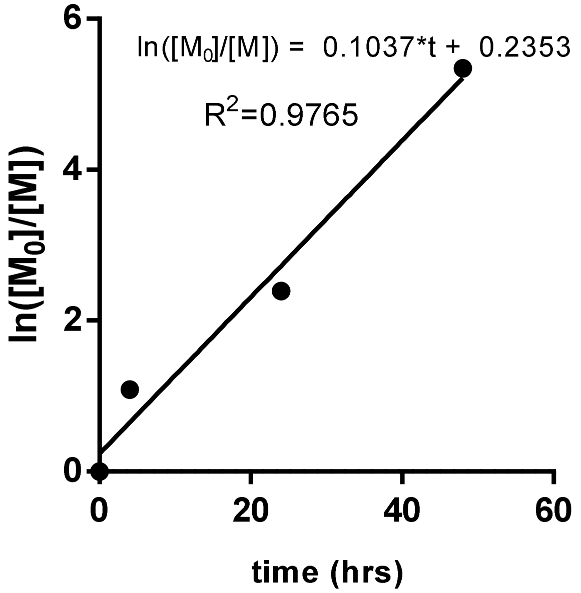

Supplementary Figure 7. First-order kinetic fit of deconvolution data of PAMK$_{63}$-*g*-PLacS$_{43}$.

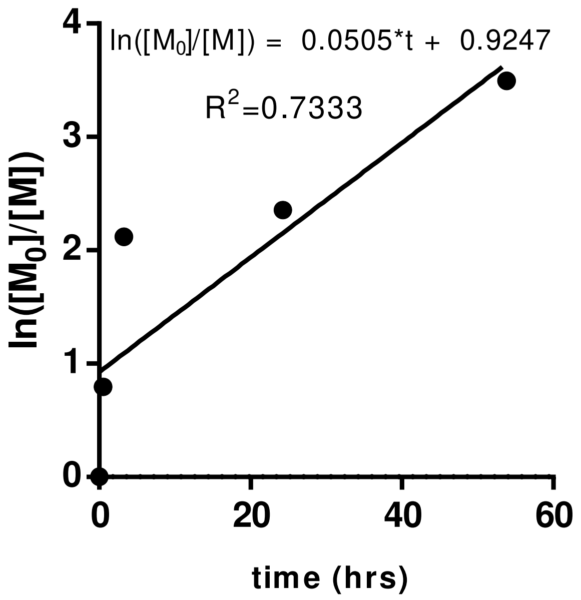

Supplementary Figure 8. First-order kinetic fit of deconvolution data of ($PAMK_{0.25}$-stat-$PZK_{0.75}$)$_{75}$-*g*-$PLacS_{25}$.

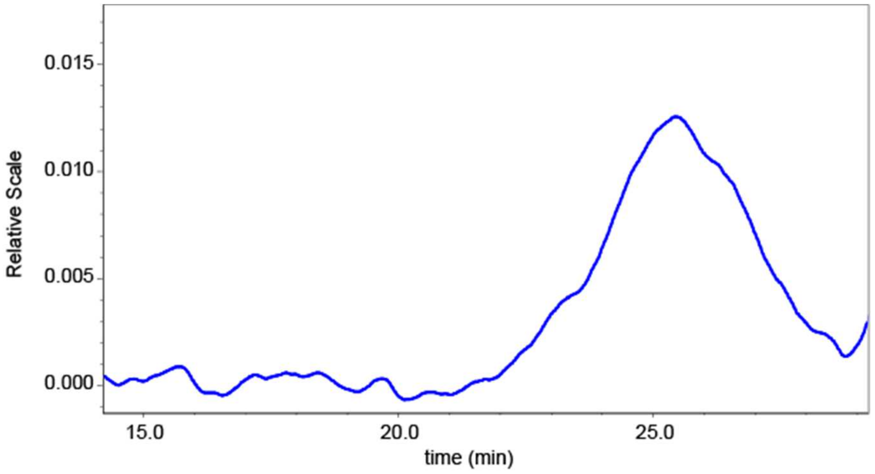

Supplementary Figure 9. Differential refractive index signal from the cleaved branches of $PAMK_{63}$-*g*-$PGalNAcS_{33}$.

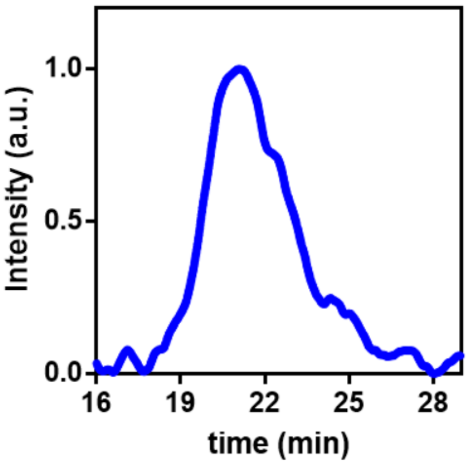

Supplementary Figure 10. Differential refractive index signal from (PtBLG$_{0.8}$-*s*-PAIK$_{0.2}$)$_{50}$-*g*-PGalNAcS$_{13}$-N$_3$

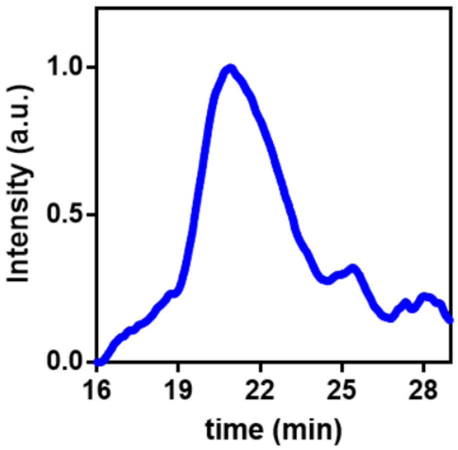

Supplementary Figure 11. Differential refractive index signal from (PtBLG$_{0.8}$-*s*-PAIK$_{0.2}$)$_{50}$-*g*-PGalNAcS$_{13}$-Chol

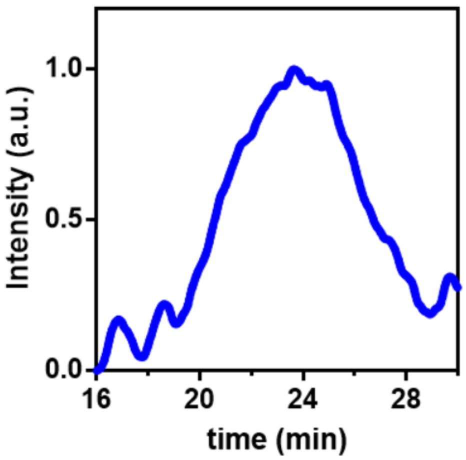

Supplementary Figure 12. Differential refractive index signal from (PtBLG$_{0.8}$-*s*-PAIK$_{0.2}$)$_{50}$- N$_3$

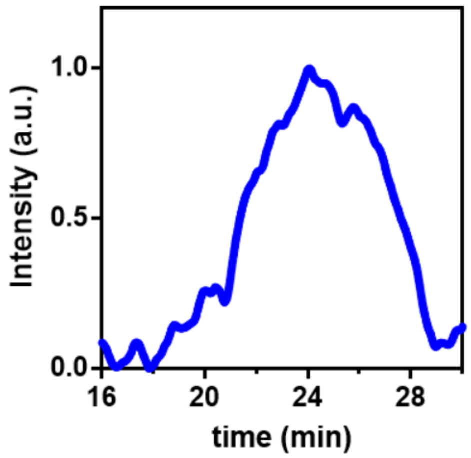

Supplementary Figure 13. Differential refractive index signal from $(PtBLG_{0.8}$-*s*-$PAIK_{0.2})_{50}$-Chol

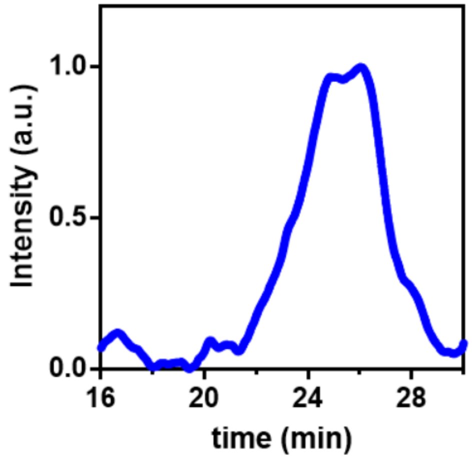

Supplementary Figure 14. Differential refractive index signal from $PAMK_{63}$

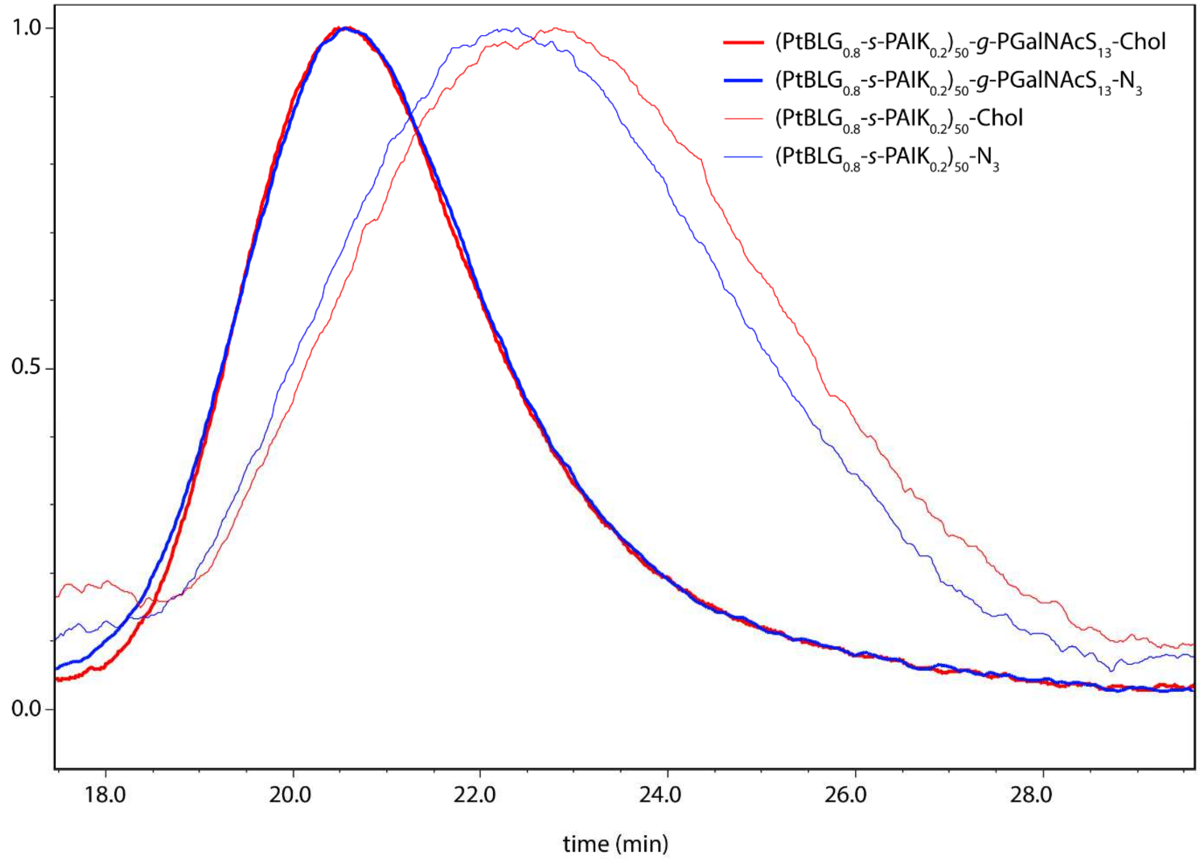

Supplementary Figure 15. GPC LS traces of the backbone and the resulting protected brushes synthesized from the same backbones used in the glycocalyx engineering experiments. tBLG is an abbreviation for tert-butoxy-L-glutamate.

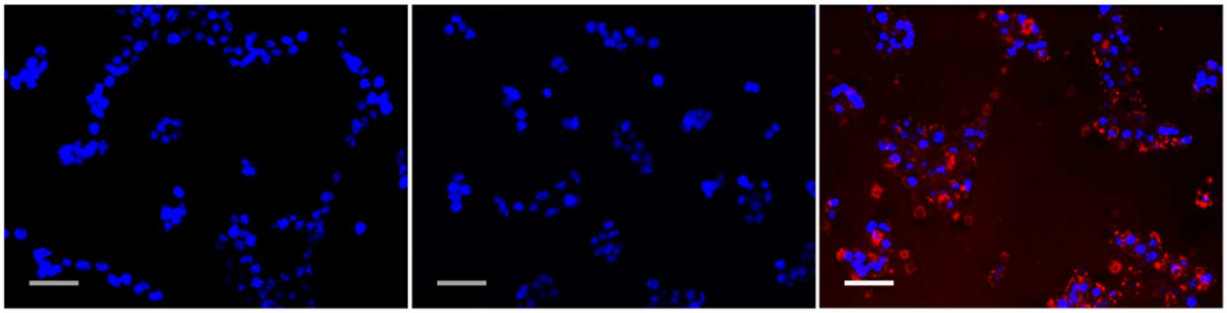

Supplementary Figure 16. Cell images in A), B), C) are 24 hours after incubation B) without polymer, C) with 15µM AF594-glycobrush-N3, or D) with 15µM AF594-glycobrush-Chol. HEK 293T cells were stained with Hoechst. Scale bar is 50 microns. Images are representative of 4 separate experiments.

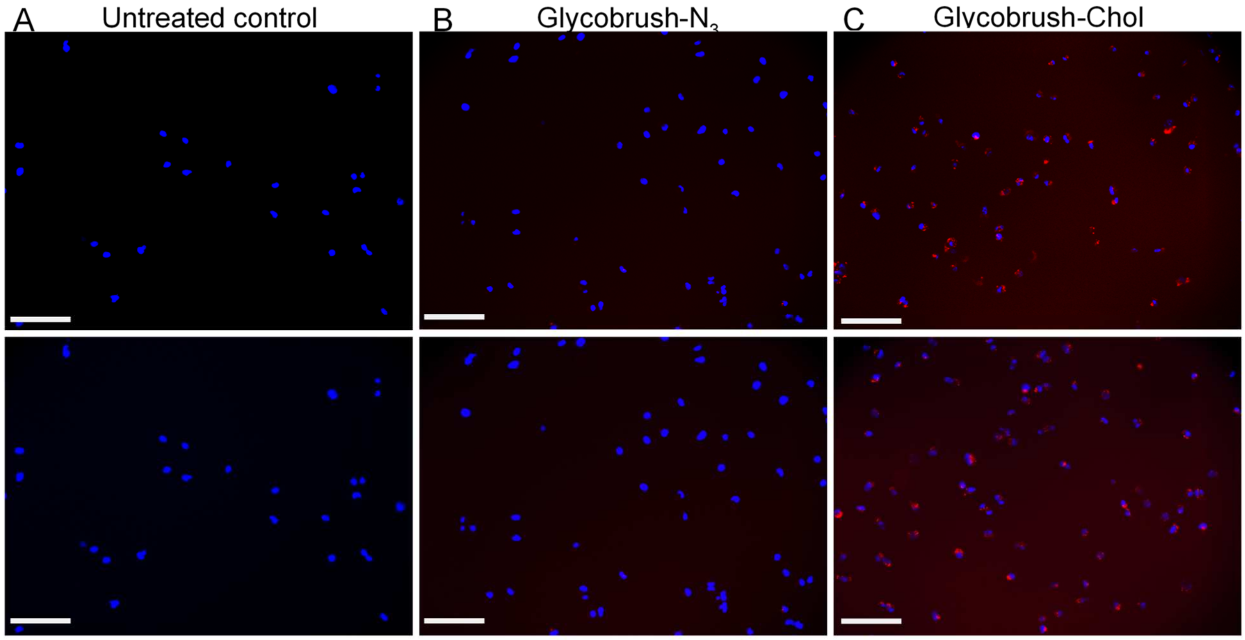

Supplementary Figure 17. Cell images in A), B), C) are 24 hours after incubation A) without polymer, B) with 15µM AF594-glycobrush-N3, or C) with 15µM AF594-glycobrush-Chol. HEK 293T cells were stained with Hoechst. Scale bar is 50 microns. Images are representative of 4 separate experiments..

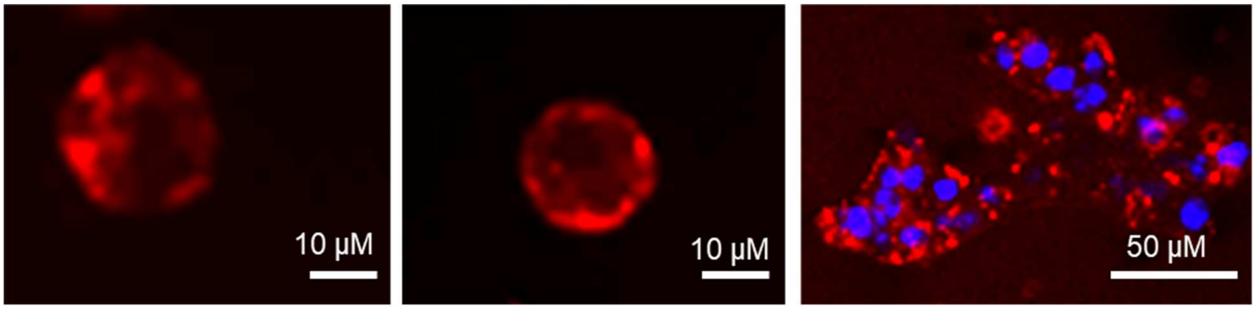

Supplementary Figure 18. Representative fluorescence images of glycocalyx engineering of HEK 293T cells treated with AF-594-(PLG$_{0.8}$-*s*-PAIK$_{0.2}$)$_{50}$-*g*-PGalNAcS$_{13}$-Chol where glycobrushes can be obersved at the cell membrane and in recycling endosomes. Left and center are single cells untreated with nuclear stain Hoescht, while right image is a cluster of cells that had been Hoechst stained. All images taken at 24 hours post-treatment. Images are representative of 4 separate experiments.

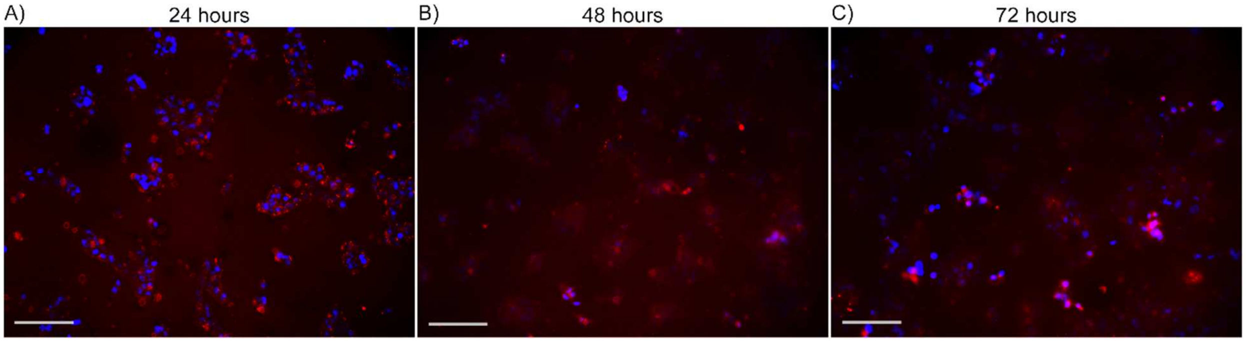

Supplementary Figure 19. Fluorescent imaging overlay of Hoechst stain and glycocalyx engineering of HEK 293T cells with AF-594-($PLG_{0.8}$-*s*-$PAIK_{0.2}$)$_{50}$-*g*-$PGalNAcS_{13}$-Chol at A) 24 hours, B) 48 hours, C) 72 hours. Scale bar is 100 microns. Images are representative of 4 separate experiments.

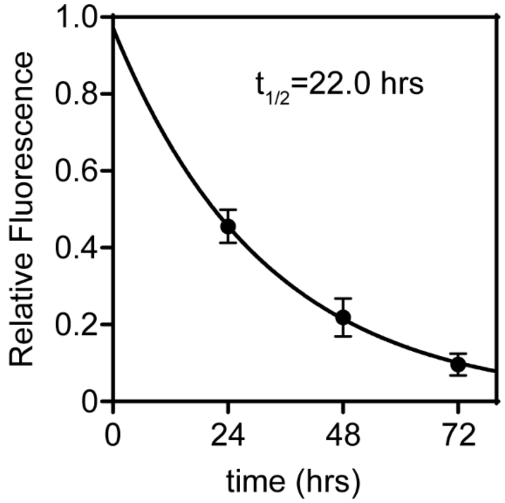

Supplementary Figure 20. Ratio of AF594 fluorescence to Hoescht fluorescence during glycocalyx engineering. An exponential fit was used to determine the half-life time for the glycobrushes on the cell surface. Average fluorescence intensity data and SEM values are as follows: 24 hr: 0.46 ± 0.043, n = 6; 48 hr: 0.22 ± 0.049, n = 3; 72 hr: 0.096 ± 0.029, n = 3.

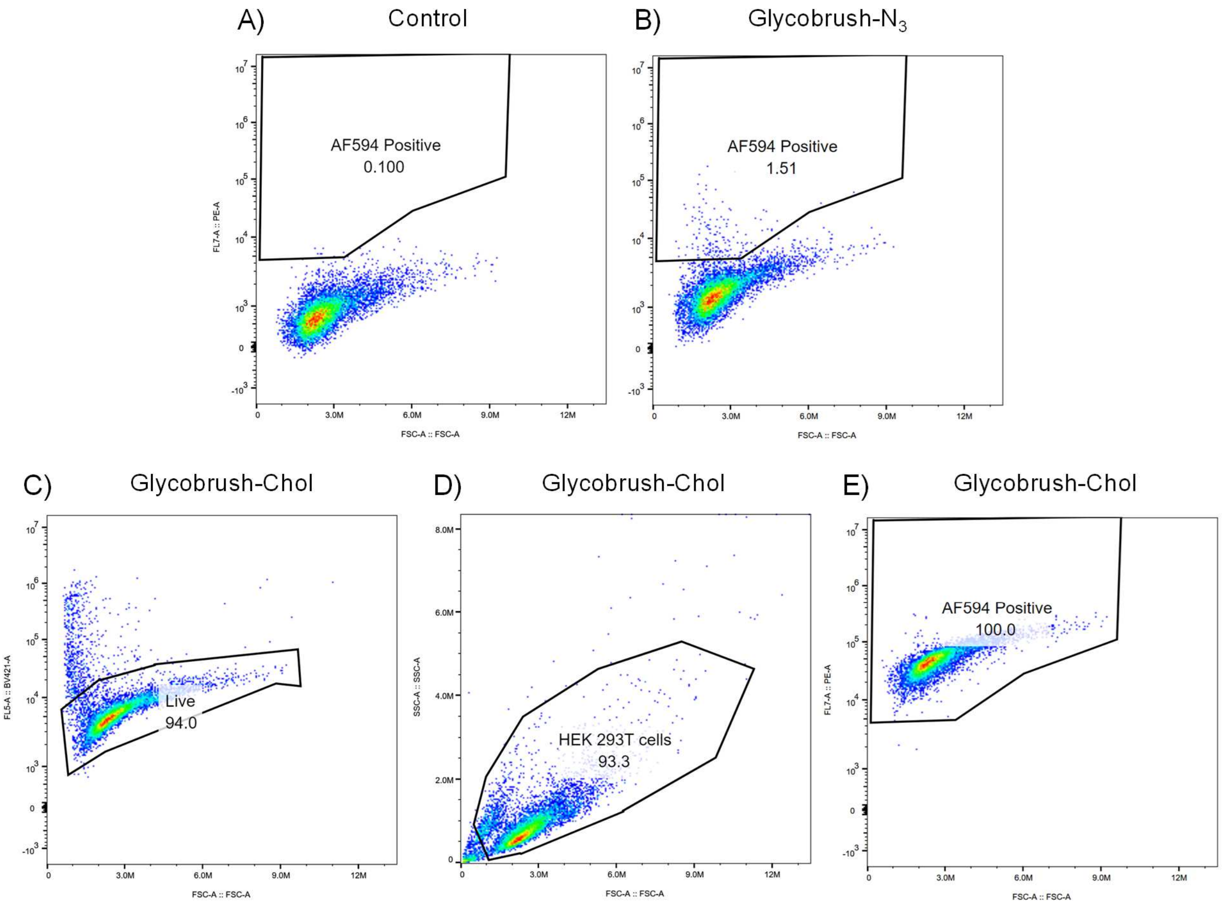

Supplementary Figure 21. Flow cytometry data gating for glycocalyx engineered HEK 293T cells where A) is control cells, B) is cells treated with AF594-glycobrush-N_3 and C-E) are cell treated with AF594-glycobrush-Chol. The entire gating tree is shown for the glycobrush-Chol samples: C) side scatter (SSC) vs. forward scatter (FSC) to exclude cell debris, D) BV421 vs. FSC to exclude dead cells positive for DAPI, and E) PE vs. FSC to identify AF594-positive cells.

IV. NMR Spectra

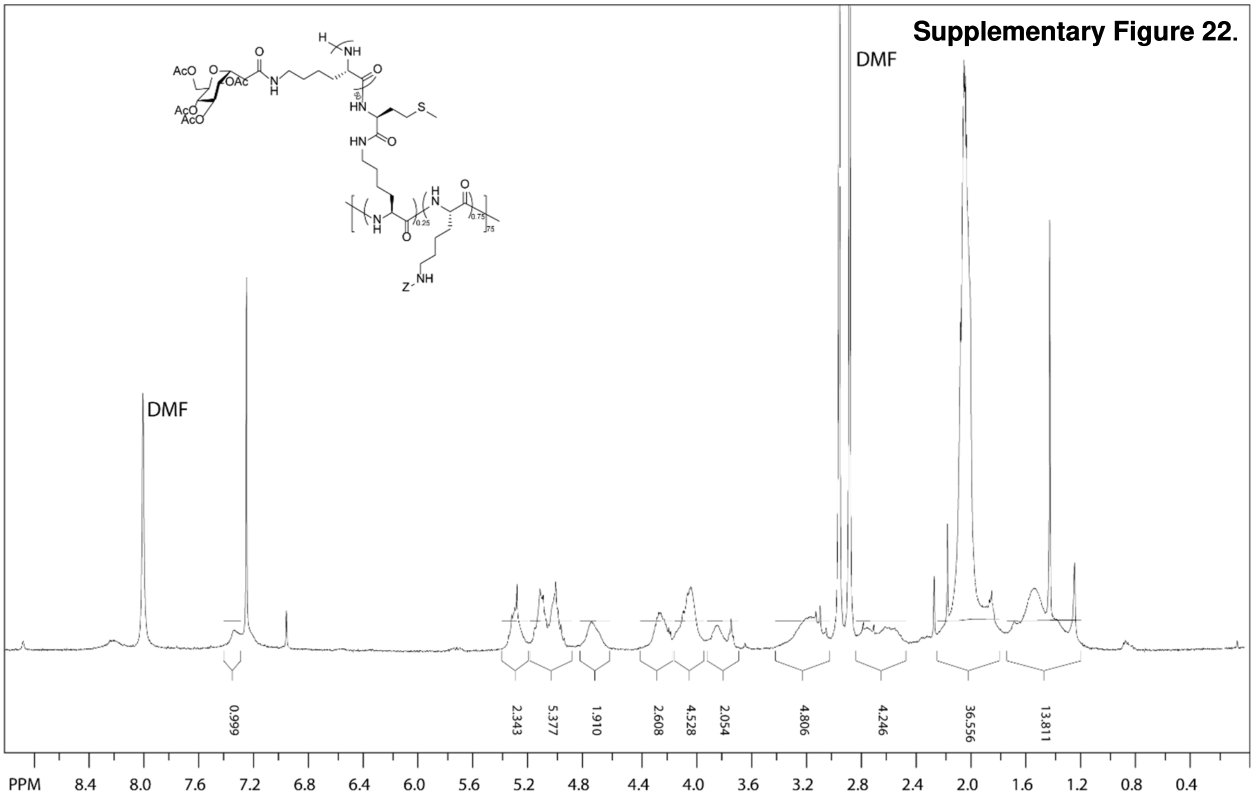

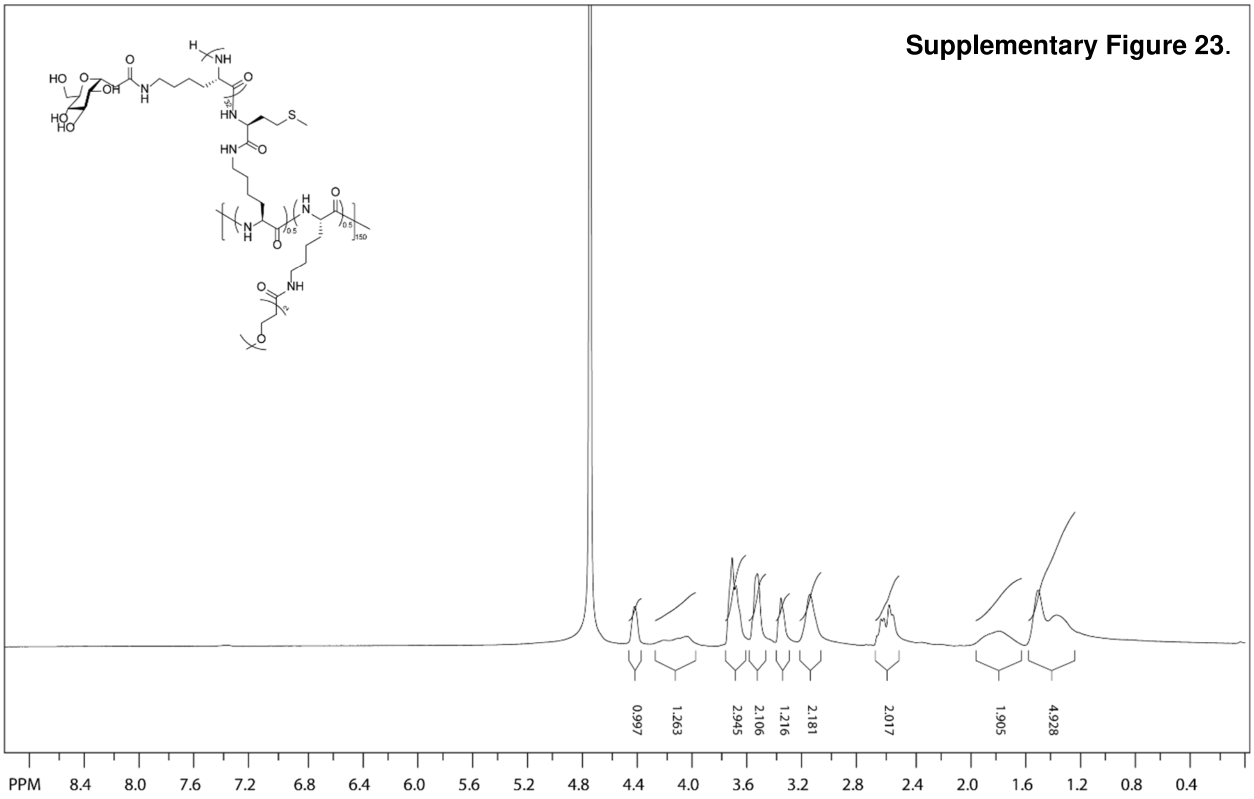

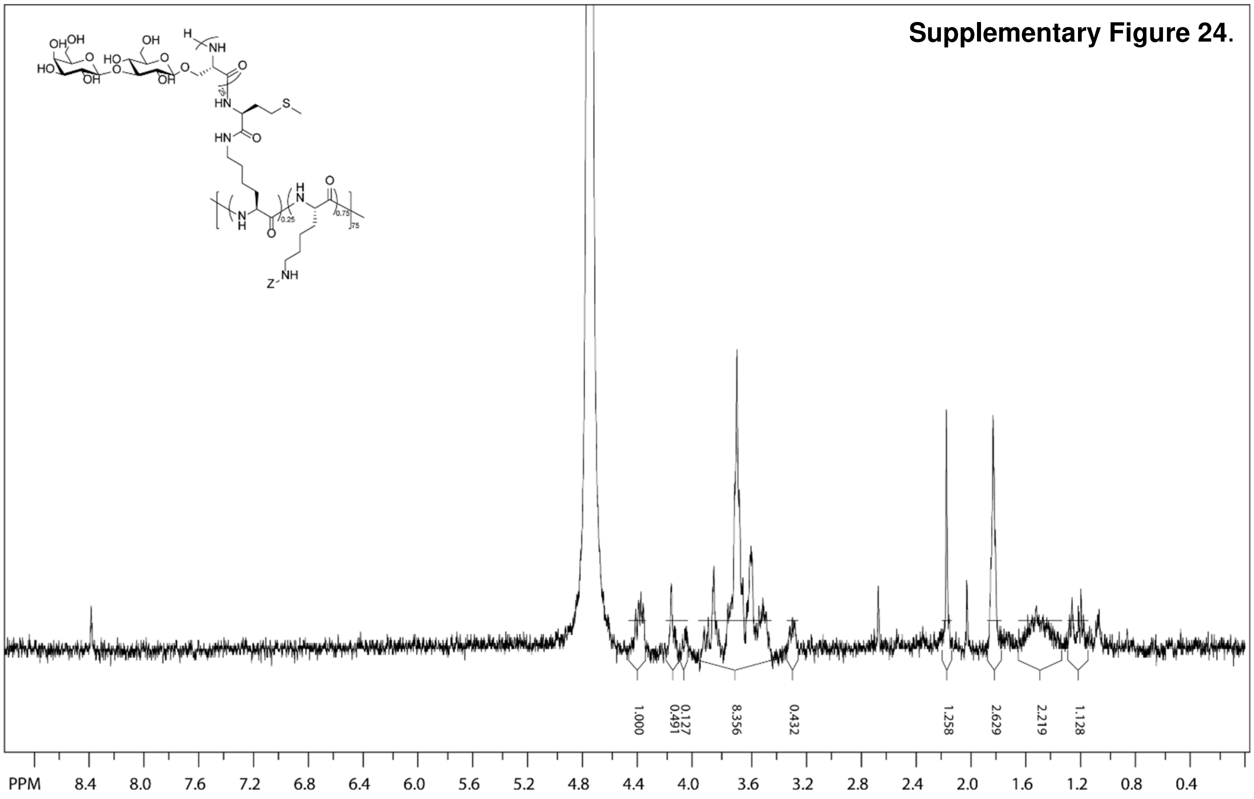

Supplementary Figure 24.

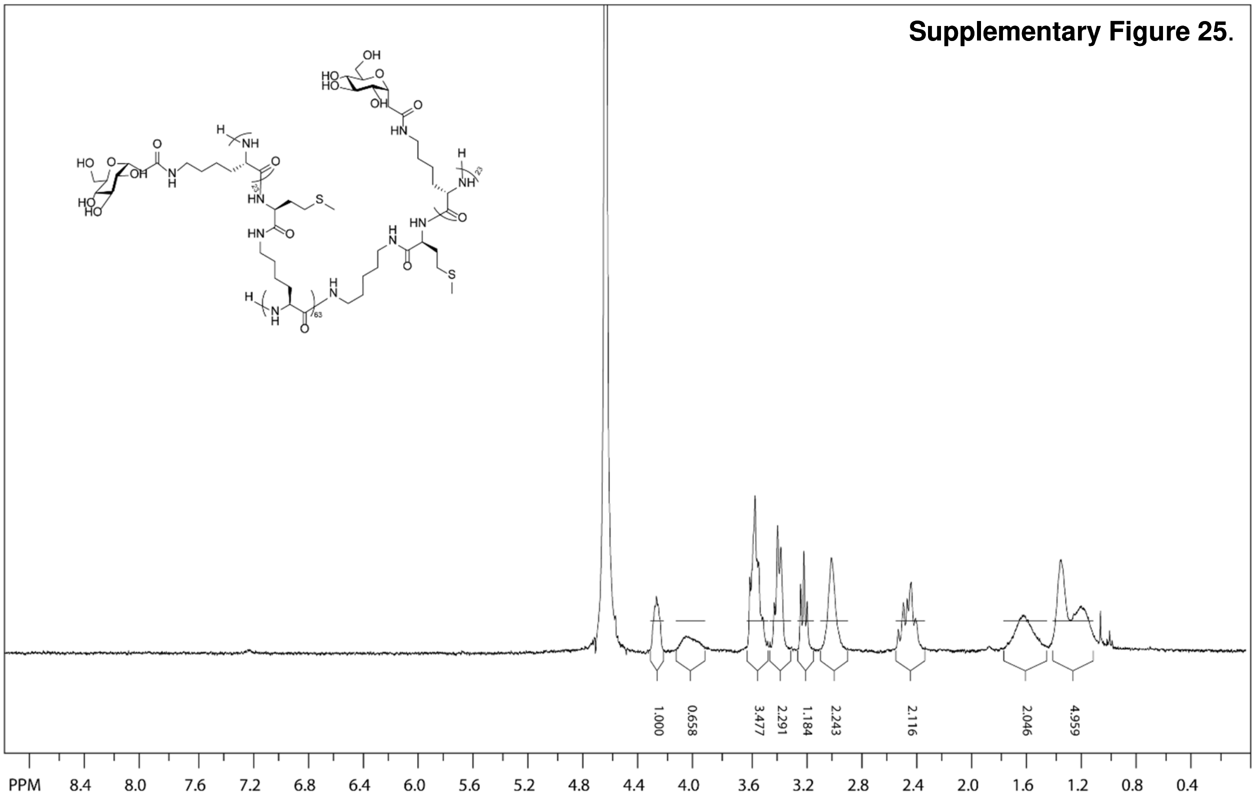

Supplementary Figure 25.

Supplementary Figure 26.

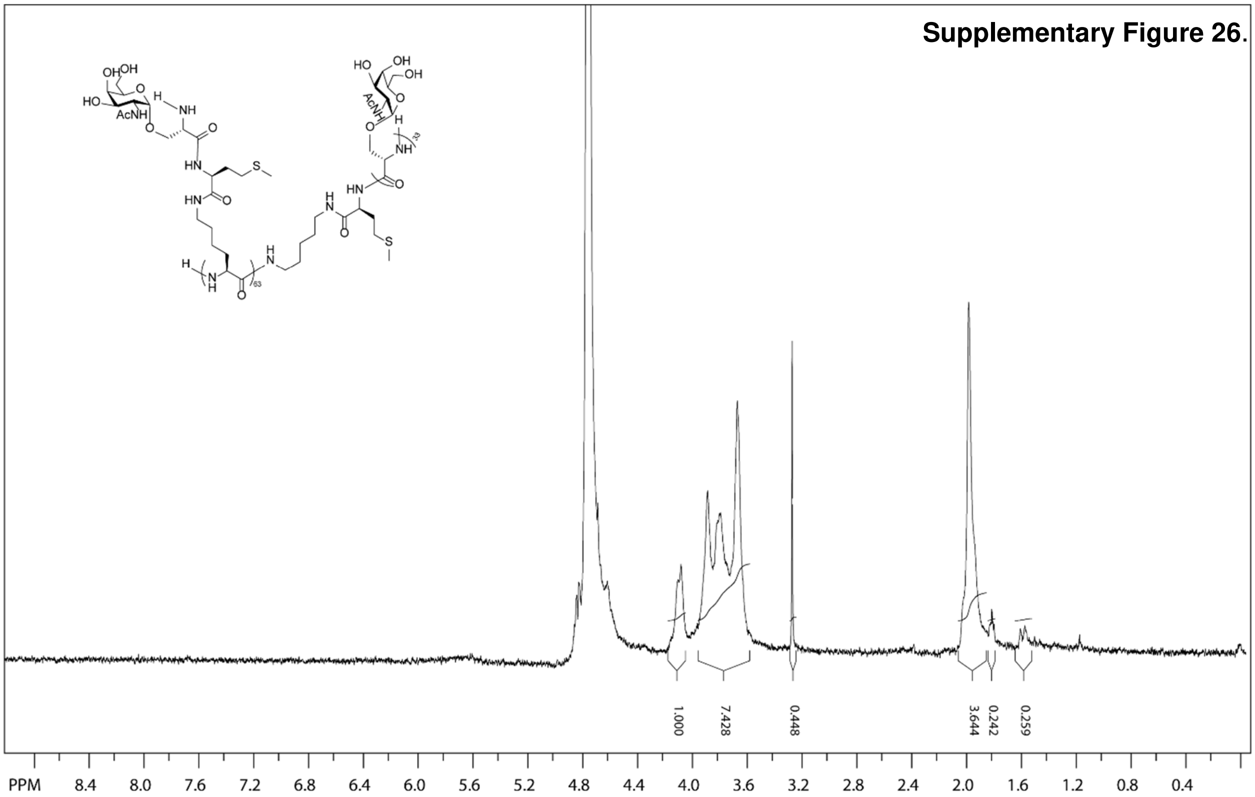

Supplementary Figure 27.

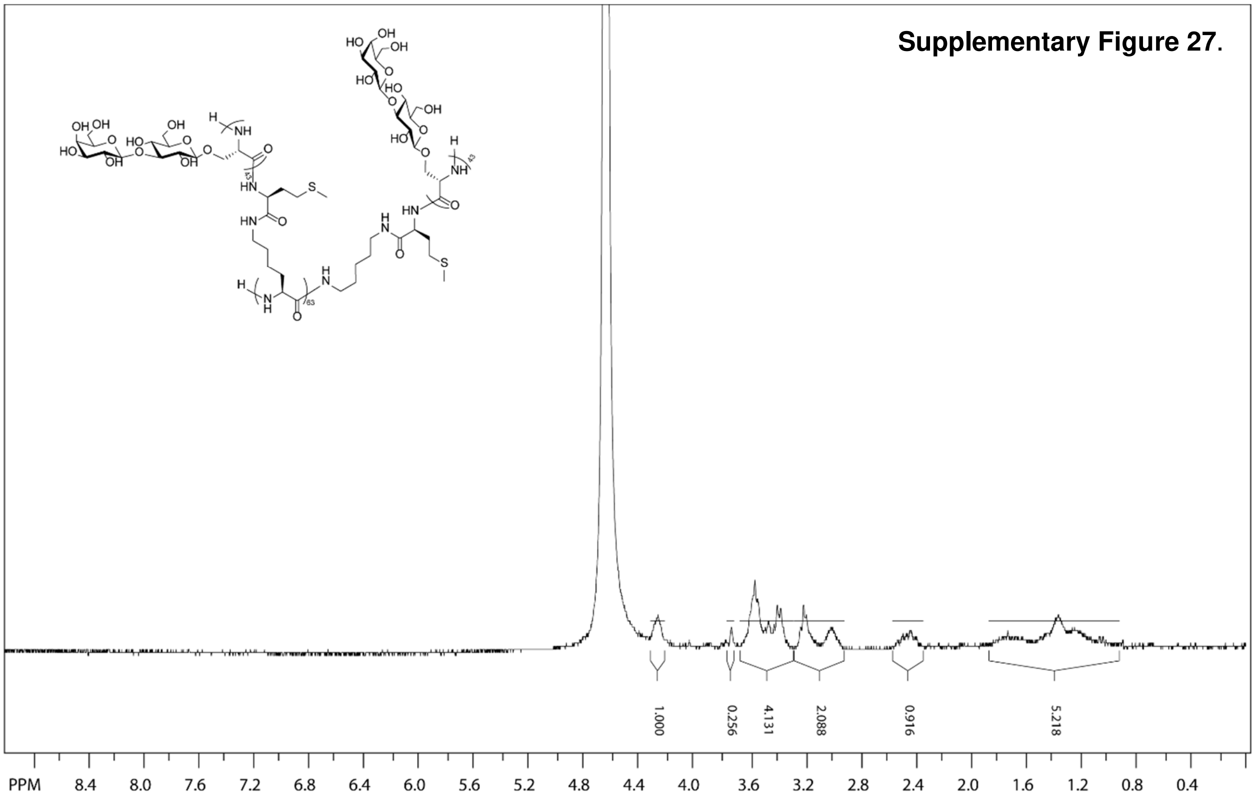

Supplementary Figure 28.

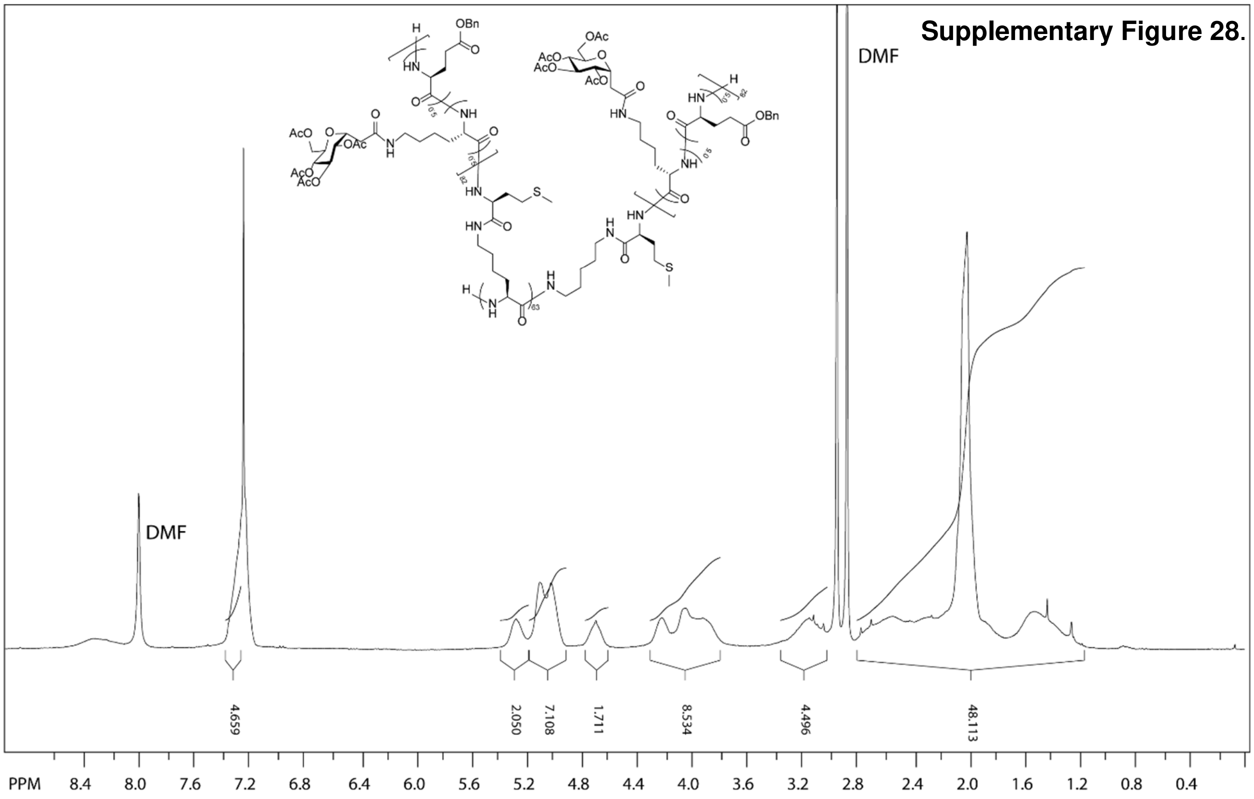

Supplementary Figure 29.

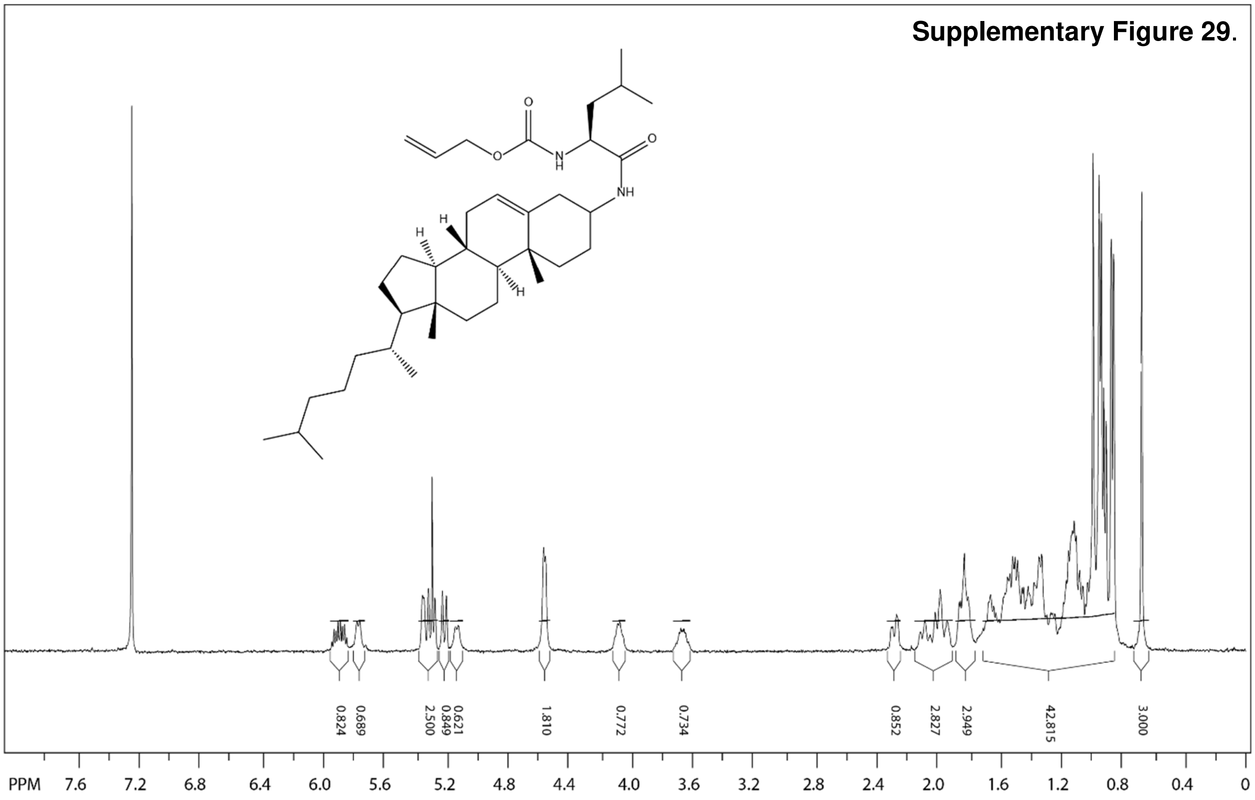

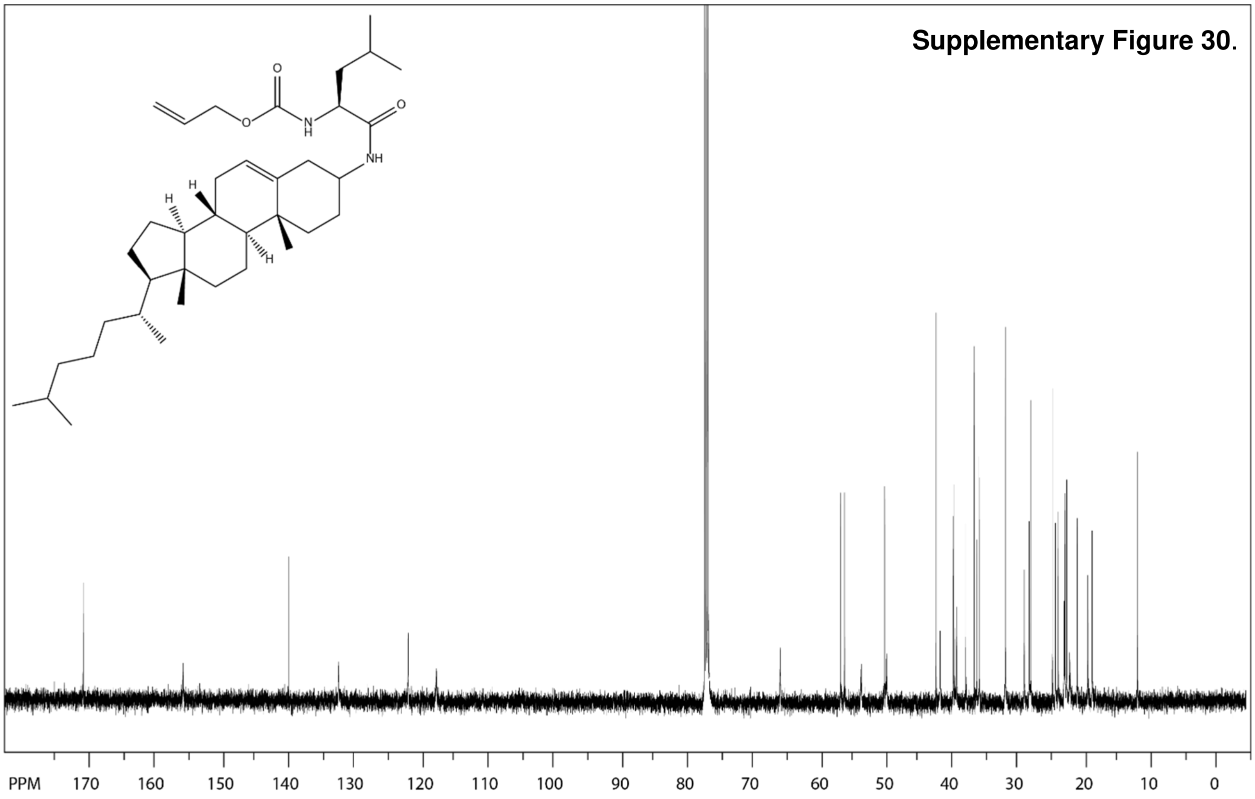

Supplementary Figure 30.

V. Supplementary References

1. Bukowski, R., Morris, L. M., Woods, R. J. & Weimar, T. Synthesis and Conformational Analysis of the T-Antigen Disaccharide. (2001).

2. Plattner, C., Hoefener, M. & Sewald, N. One-Pot Azidochlorination of Glycals. *Org. Lett.* **42**, no-no (2011).

3. Kramer, J. R., Onoa, B., Bustamante, C. & Bertozzi, C. R. Chemically tunable mucin chimeras assembled on living cells. *Proc. Natl. Acad. Sci.* **112**, 12574–12579 (2015).

4. Kramer, J. R. & Deming, T. J. Glycopolypeptides via Living Polymerization of Glycosylated- L-lysine N -Carboxyanhydrides. *J. Am. Chem. Soc.* **132**, 15068–15071 (2010).

5. Merbouh, N., Wallner, F. K., Cociorva, O. M. & Seeberger, P. H. 3-Mercaptopropanol as a traceless linker for chemical and enzymatic synthesis of oligosaccharides. *Org. Lett.* **9**, 651–653 (2007).

6. Zhou, M. N. *et al.* N-Carboxyanhydride Polymerization of Glycopolypeptides That Activate Antigen-Presenting Cells through Dectin-1 and Dectin-2. *Angew. Chemie - Int. Ed.* **12**, 3137–3142 (2018).

7. Rhodes, A. J. & Deming, T. J. Tandem catalysis for the preparation of cylindrical polypeptide brushes. *J. Am. Chem. Soc.* **134**, 19463–19467 (2012).

8. Kramer, J. R. & Deming, T. J. General Method for Purification of α-Amino acid- N -carboxyanhydrides Using Flash Chromatography. *Biomacromolecules* **11**, 3668–3672 (2010).

9. Sun, Q., Cai, S. & Peterson, B. R. Practical synthesis of 3β-amino-5-cholestene and related 3β-halides involving i-steroid and retro-i-steroid rearrangements. *Org. Lett.* (2009). doi:10.1021/ol802343z

10. Newville, Matthew; Stensitzki, Till; Allen, Daniel B.; Ingargiola, A. LMFIT: Non-linear Least-square Minimization and Curve-fitting for Python. *Zenodo* (2014). doi:10.5281/zenodo.598352

11. Atkins, P. W. P. W. Atkins: Physical Chemistry, 4th Edition, Oxford University Press, Oxford, ISBN 0-19-855284-X, 1990. 995 Seiten, Preis: £ 19.50 (Paperback). *Berichte der Bunsengesellschaft für Phys. Chemie* **94**, 1171–1171 (1990).

12. Malaker, S. A. *et al.* The mucin-selective protease StcE enables molecular and functional analysis of human cancer-associated mucins. *Proc. Natl. Acad. Sci.* **116**, 7278 LP – 7287 (2019).
