# Supplementary material for: Tunable, biodegradable grafting-from glycopolypeptide bottlebrush polymers
Source: Nat Commun. 2021 Nov 9;12:6472. doi: 10.1038/s41467-021-26808-5 (PMC8578664; doi:10.1038/s41467-021-26808-5)
Supplement: Supplementary file 2 — Reporting Summary [file 41467_2021_26808_MOESM2_ESM.pdf]

nature portfolio

Corresponding author(s): Jessica R. Kramer

Last updated by author(s): Sep 15, 2021

Reporting Summary

Nature Portfolio wishes to improve the reproducibility of the work that we publish. This form provides structure for consistency and transparency in reporting. For further information on Nature Portfolio policies, see our Editorial Policies and the Editorial Policy Checklist.

Statistics

For all statistical analyses, confirm that the following items are present in the figure legend, table legend, main text, or Methods section.

n/a	Confirmed	
☐	☒	The exact sample size (*n*) for each experimental group/condition, given as a discrete number and unit of measurement
☐	☒	A statement on whether measurements were taken from distinct samples or whether the same sample was measured repeatedly
☐	☒	The statistical test(s) used AND whether they are one- or two-sided<br>*Only common tests should be described solely by name; describe more complex techniques in the Methods section.*
☐	☒	A description of all covariates tested
☐	☒	A description of any assumptions or corrections, such as tests of normality and adjustment for multiple comparisons
☐	☒	A full description of the statistical parameters including central tendency (e.g. means) or other basic estimates (e.g. regression coefficient) AND variation (e.g. standard deviation) or associated estimates of uncertainty (e.g. confidence intervals)
☐	☒	For null hypothesis testing, the test statistic (e.g. *F*, *t*, *r*) with confidence intervals, effect sizes, degrees of freedom and *P* value noted<br>*Give P values as exact values whenever suitable.*
☒	☐	For Bayesian analysis, information on the choice of priors and Markov chain Monte Carlo settings
☐	☒	For hierarchical and complex designs, identification of the appropriate level for tests and full reporting of outcomes
☒	☐	Estimates of effect sizes (e.g. Cohen's *d*, Pearson's *r*), indicating how they were calculated

Our web collection on statistics for biologists contains articles on many of the points above.

Software and code

Policy information about availability of computer code

Data collection	Astra 7.3.0 was used to collect refractive and light scattering index data from Wyatt OptiLab REx and DAWN Helios instruments. OPUS 8.5 was used to collect FTIR data on a Bruker Alpha instrument. DLS was acquired via Malvern Panalytical Zetasizer Software version 7.13. m Nano ZS. Absorbance data was acquired on a Nanodrop 2000 made by ThermoFisher using software Nanodrop 2000 Version V3. Cytotox data was acquired on a plate reader by Molecular Devices Spectramax M2 using Softmax Pro 6.1.
Data analysis	FTIR data was analyzed with OPUS 7.5 and for SEC MALS/RI Astra 7.3.0. Flow cytometry data was analyzed using Flow Jo v10.8.0. Gwyddion v2.53 was used to process AFM images and RStudio v3.6.1 was used to create the violin plot. GraphPad Prism version 6, Matlab 2016A, and python 3.7 were used for calculations, data deconvolution, and plot preparation. ImageJ version 1.51j8 was used for image analysis.

For manuscripts utilizing custom algorithms or software that are central to the research but not yet described in published literature, software must be made available to editors and reviewers. We strongly encourage code deposition in a community repository (e.g. GitHub). See the Nature Portfolio guidelines for submitting code & software for further information.

Data

Policy information about availability of data

All manuscripts must include a data availability statement. This statement should provide the following information, where applicable:

- Accession codes, unique identifiers, or web links for publicly available datasets
- A description of any restrictions on data availability
- For clinical datasets or third party data, please ensure that the statement adheres to our policy

All data related to key findings are presented within the manuscript and supplementary information. Raw data files are available upon request. N/A regarding use of public data sets, public repositories, clinical data, or third party data.

Field-specific reporting

Please select the one below that is the best fit for your research. If you are not sure, read the appropriate sections before making your selection.

☒ Life sciences ☐ Behavioural & social sciences ☐ Ecological, evolutionary & environmental sciences

For a reference copy of the document with all sections, see nature.com/documents/nr-reporting-summary-flat.pdf

Life sciences study design

All studies must disclose on these points even when the disclosure is negative.

Sample size	Flow cytometry was performed on 10000 cells at minimum. Cellular experiments were performed in 3-6 replicates and glycocalyx engineering experiments were repeated in 4 separate experiment sets. Sample sizes were chosen to provide sufficient data for analysis with standard error measurement or Mann-Whitney test. Sample sizes were chosen without sample size determination calculations.
Data exclusions	no data were excluded
Replication	all reported data was reproducible in >3 experimental sets
Randomization	No animals or humans were used in this study. For cellular work randomization is not applicable as all data was collected with the same instrument settings regarding both flow cytometry and imaging fluorescence.
Blinding	No animals or humans were used in this study. For cellular work blinding is not applicable as all data was collected with the same instrument settings regarding both flow cytometry and imaging fluorescence.

Reporting for specific materials, systems and methods

We require information from authors about some types of materials, experimental systems and methods used in many studies. Here, indicate whether each material, system or method listed is relevant to your study. If you are not sure if a list item applies to your research, read the appropriate section before selecting a response.

Materials & experimental systems

n/a	Involved in the study	
☒	☐	Antibodies
☐	☒	Eukaryotic cell lines
☒	☐	Palaeontology and archaeology
☒	☐	Animals and other organisms
☒	☐	Human research participants
☒	☐	Clinical data
☒	☐	Dual use research of concern

Methods

n/a	Involved in the study	
☒	☐	ChIP-seq
☐	☒	Flow cytometry
☒	☐	MRI-based neuroimaging

Eukaryotic cell lines

Policy information about cell lines

Cell line source(s)	ATCC, HEK293T
Authentication	The cell lines were purchased from ATCC and were not authenticated further.
Mycoplasma contamination	cells tested negative for mycoplasma
Commonly misidentified lines (See ICLAC register)	none known

Flow Cytometry

Plots

Confirm that:

☒ The axis labels state the marker and fluorochrome used (e.g. CD4-FITC).

☒ The axis scales are clearly visible. Include numbers along axes only for bottom left plot of group (a 'group' is an analysis of identical markers).

☒ All plots are contour plots with outliers or pseudocolor plots.

☒ A numerical value for number of cells or percentage (with statistics) is provided.

Methodology

Sample preparation	HEK 293T cells were acquired from ATCC. AF594-labeled glycopolypeptides were dissolved at 15 µM in complete media (DMEM with 10% FBS, pen/strep, and L-glutamine) and sterile-filtered through a 0.2 µm membrane. HEK293T cells were trypsinized and neutralized with complete media. Cells were pelleted by centrifugation at 200xg for 5 minutes. Media was removed and cells were resuspended in media containing polymer. Cells were then incubated in the media + polymer for 2 hours at room temperature. Incubation could be conducted in the microcentrifuge tube, but transfer to a culture plate was preferred for improved surface area. Cells did not adhere at room temperature. Post incubation, treated cells were resuspended and centrifuged, washed with PBS, resuspended in complete media (lacking polymer), and plated. Mock-engineered control cells were plated on a separate 24-well plate. All cells were left to grow at 37 °C. After the PBS wash outlined above, mock engineered and treated cells were resuspended in PBS at ~106 cells/mL. DAPI was added as a live/dead discriminator.
Instrument	Data was acquired on a Beckman Coulter Cytoflex S flow cytometer
Software	Data was analyzed using Flow Jo v10.8.0
Cell population abundance	Of the glycobrush-Chol treated cells, 100% were AF594-positive as compared to only 1.5% of the glycobrush-N3 -treated cells and only 0.3% of the mock treated control. Chol-terminal glycobrushes were observed on the cell-surface for up to 3 days with a half-life of 22 hrs.
Gating strategy	The gating tree was as follows: 1) FSC/SSC to 2) live gate (DAPI negative) to 3) SSC/PE (AF594 positive). See SI Fig S21 for gating.

☒ Tick this box to confirm that a figure exemplifying the gating strategy is provided in the Supplementary Information.
